# Effects of climate change and land cover on the distributions of a critical tree family in the Philippines

**DOI:** 10.1038/s41598-020-79491-9

**Published:** 2021-01-11

**Authors:** Sean E. H. Pang, Jose Don T. De Alban, Edward L. Webb

**Affiliations:** Department of Biological Sciences, National University of Singapore, Singapore, 117543 Singapore

**Keywords:** Climate-change impacts, Biodiversity, Ecological modelling, Tropical ecology, Forest ecology, Macroecology

## Abstract

Southeast Asian forests are dominated by the tree family Dipterocarpaceae, whose abundance and diversity are key to maintaining the structure and function of tropical forests. Like most biodiversity, dipterocarps are threatened by deforestation and climate change, so it is crucial to understand the potential impacts of these threats on current and future dipterocarp distributions. We developed species distribution models (SDMs) for 19 species of dipterocarps in the Philippines, which were projected onto current and two 2070 representative concentration pathway (RCP) climate scenarios, RCP 4.5 and 8.5. Current land cover was incorporated as a post-hoc correction to restrict projections onto intact habitats. Land cover correction alone reduced current species distributions by a median 67%, and within protected areas by 37%. After land cover correction, climate change reduced distributions by a median 16% (RCP 4.5) and 27% (RCP 8.5) at the national level, with similar losses in protected areas. There was a detectable upward elevation shift of species distributions, consisting of suitable habitat losses below 300 m and gains above 600 m. Species-rich stable areas of continued habitat suitability (i.e., climate macrorefugia) fell largely outside current delineations of protected areas, indicating a need to improve protected area planning. This study highlights how SDMs can provide projections that can inform protected area planning in the tropics.

## Introduction

Biodiversity loss worldwide is proceeding at unprecedented rates^1^. Among the major drivers of biodiversity loss, anthropogenic land use (i.e., habitat loss) and climate change are recognised as the most urgent threats^2–4^. Land cover change immediately reduces the extent of suitable habitat for resident species^2,5^ and has second-order implications for species persistence in fragmented landscapes such as reduced habitat connectivity^6,7^. Similarly, climate change has long-term implications for species, with predicted alterations to temperature, precipitation and seasonality leading to concomitant shifts in species distribution^3,8^. Indeed, both field-based historical studies^9,10^ and climate change informed projections of plant species distributions indicate shifts and changes in distribution^3,8,11,12^. Distribution assessments through the use of species distribution models (SDM) could estimate the risk these drivers pose to species, better-asserting species vulnerabilities^13–15^.

Southeast Asia is a hotspot of both biodiversity and deforestation^16,17^, and is expected to be heavily impacted by climate change^8,18^. The Philippine archipelago is particularly emblematic of the threats to Southeast Asia (SEA), being a biodiversity hotspot with a large number of endemics but which is also biologically undersurveyed^19^, resulting in a lack of contemporary occurrence records^20,21^. The Philippines has also experienced intensive logging and agricultural expansion, leaving only 3% of its primary forests intact^22,23^, and is exceptionally vulnerable to climate change^18^. In this regard, as the dominant family of trees in SEA forests, species from the family Dipterocarpaceae are of exceptional interest^24–26^. These non-pioneering, often fast-growing and highly productive trees are amongst the tallest in the tropics, and comprise the majority of above-ground carbon stores and emergent individuals^24,26–28^. With high functional variation and effective exploitation of different environmental niches, their abundance and diversity are key to maintaining the structure and function of tropical forests^24,27–29^. Considering the significance of dipterocarps to forests in the Philippines^24–26^, shifts in their distribution are likely to affect the integrity of ecosystems and its constituent species^29–33^. It is of great importance, therefore, to understand the potential effects of anthropogenic land use and climate change on dipterocarp distributions in the Philippines to better conserve remaining forests^27,34–36^.

A potential mitigation measure against these threats is protected areas^37,38^. Protected areas provide safe havens for species, potentially preventing habitat loss from anthropogenic land use or as buffers against the changing climate^37–39^. Thus, the coverage afforded by protected areas and the distribution changes within them are important for distribution assessments and conservation efforts.

Areas of continued habitat suitability under climate change are similarly important, whose availability is especially crucial in safeguarding the persistence of species with limited dispersal abilities such as dipterocarps^34,39^. Part of meeting the challenges of climate change would be to ensure that these areas are protected or prioritised for protection^34,40^.

It is widely recognised that land cover should be included as a spatially-explicit variable in SDMs^41,42^, and is crucial for distribution assessments^36,43^. Incorporating land cover as a variable into a SDM—the most common practice—generates new, restrictive, requirements for the use of species occurrence data^44^. Specifically, the land cover data being integrated into SDMs is often contemporary, implying that occurrence data should be correspondingly recent to allow for accurate occurrence-land cover associations. Temporal mismatches between historical species occurrences and contemporary land cover may pose constraints for SDMs by inaccurately assigning species-habitat associations, in cases where the land cover has changed after the date of occurrence data collection (e.g., a collection made in a forest before it was converted to agricultural land). This is an important consideration for tropical developing countries such as in Southeast Asia, which have experienced rapid rates of deforestation^16,17^, but which also tend to have high biodiversity and be poorly biologically surveyed^5,20^. Nevertheless, occurrence points located on changing landscapes remain important representations of species’ environmental niches and should be included where possible. Simply excluding occurrences mismatched against contemporary land cover would likely introduce sampling biases and niche truncations, biasing SDM estimates^45–47^. In this study, we investigated the effects of anthropogenic land use and climate change on the current and future distributions of dipterocarps in the Philippines. Our study applied the well-established maximum entropy modelling approach (MaxEnt; Phillips et al.^15^) to model species distributions for the current and two 2070 climate scenarios. Our specific objectives were to assess the effects of land cover and climate change on the distribution of dipterocarps within and outside of protected areas, and subsequently to infer the adequacy of protection currently afforded to areas of continued habitat suitability.

## Materials and methods

### Study area

The Philippines is the world’s second-largest archipelago, consisting of more than 7600 islands covering about 300,000 km^2^. The two largest islands of Luzon and Mindanao constitute two-thirds of the total land area. The topography of the country varies greatly and ranges from sea level to 2954 m above sea level (asl) (Mt. Apo, Mindanao Island). The tropical climate is influenced by the southwest and northeast monsoons, characterised by a wet season from June to November and a dry season from December to May. The mean annual rainfall varies from 965 to 4064 mm across the country^48^. The mean monthly temperature ranges from 26 to 28 °C for warmer months (March to October) and from 25 to 26 °C for the cooler months (November to February). The coldest month is January (mean = 25.5 °C) while the warmest month is May (mean = 28.3 °C)^48^.

### Data acquisition and preparation

Occurrence data for Dipterocarpaceae species were gathered from the Global Biodiversity Information Facility (GBIF)^49^, and a cleaned, filtered, and verified georeferenced database of Philippine tree species published by Ramos et al.^21^ (Table 1). Observations from GBIF were filtered to only include observations with spatial accuracy comparable to the resolution of the variables used, and importantly, were not limited to the Philippines in order to better model the species’ fundamental niche, reducing the risk of niche truncations^46,50^. Almost all selected species are threatened globally and locally in the Philippines^51,52^. Only known native dipterocarps of the Philippines with at least one occurrence point within the Philippines, and at least 10 in total, were retained. Single island endemics were excluded^50^. Occurrence points for each species were then thinned at 10 km to address spatial sampling bias^53–55^. Most observations were fairly recent (> 1960), although several stretched as far back as 1902. While this could lead to temporal mismatches with the contemporary land cover map, we avoided this by applying land cover *post-hoc* (see below). The data represented 26 of 54 species, and 5 of 6 genera of dipterocarps in the Philippines, of which, 19 species and 4 genera were eventually assessed (Table 1)^56^. Nomenclature follows The International Plant Names Index^57^.

**Table 1:** The final 19 Dipterocarpaceae species in the Philippines included in this study showing the number of occurrences remaining after thinning (N), their threat status based on DAO 2017–11 and IUCN 2019 (DAO^51^; IUCN^52^), their habitats (Fernando et al.^26^; Pelser et al.^56^), and if they are a dominant species (Fernando et al.^26^).

Species	N	DAO^a^	IUCN^a^	Habitats/Forest formations	Dominant
*Dipterocarpus gracilis* Blume	111	VU	CR	Seasonal semi-evergreen rainforest, well-drained soil. Up to 800 m	
*Dipterocarpus grandiflorus* (Blanco) Blanco	109	VU	CR	Semi-evergreen rainforest, exposed ridges. Up to 500 m	✓
*Dipterocarpus hasseltii* Blume	56	VU	CR	Lowland evergreen rainforest, forest over limestone. Up to 1,000 m	
*Dipterocarpus kunstleri* King	44	VU	CR	Lowland forests, on undulating or flat land, especially near streams	
*Dipterocarpus validus* Blume	27	ND	CR	Lowland evergreen rainforest, lower slopes, plains near streams, low hills. Up to 300 m	
*Hopea acuminata* Merr	32	EN	CR	Lowland evergreen, semi-evergreen rainforest, forest over limestone. 100–800 m	
*Hopea malibato* Foxw	31	CR	CR	Non-seasonal lowland evergreen rainforest, well-drained soil. Up to 700 m	
*Hopea plagata* (Blanco) S. Vidal	21	VU	CR	Lowland evergreen and seasonal semi-evergreen rainforest, forest over limestone	
*Shorea almon* Foxw	45	VU	CR	Lowland evergreen and lower montane rainforest. Up to 1,100 m	✓
*Shorea assamica* Dyer	21	ND	LC	Lowland evergreen and semi-evergreen forests in seasonal and relatively non-seasonal regions	
*Shorea astylosa* Foxw	12	CR	CR	Primary lowland evergreen dipterocarp forest. Lower elevations	
*Shorea contorta* S. Vidal	63	VU	CR	Lowland evergreen, semi-evergreen, lower montane rainforest, forest over limestone Up to 1,000 m	✓
*Shorea guiso* (Blanco) Blume	108	ND	CR	Lowland evergreen and semi-evergreen rainforest, forest over limestone	
*Shorea hopeifolia* (F. Heim)	36	ND	CR	Primary forests on fertile and clay rich soil, with undulating terrain and hills below 600 m	
*Shorea ovata* Dyer ex Brandis	40	EN	EN	Primary forests at medium elevations up to 900 m	
*Shorea palosapis* (Blanco) Merr	43	ND	CR	Lowland evergreen rainforest on fertile, well-drained soil. Lower montane rainforest up to 1,000 m but more common in lower elevations	✓
*Shorea polysperma* (Blanco) Merr	58	VU	CR	Lowland evergreen and lower montane rainforest, forest over limestone. Up to 1,100 m	✓
*Shorea virescens* Parijs	24	ND	ND	Primary lowland evergreen forests on fertile well-drained soil. Flat and undulating terrains, and low hills up to 500 m	
*Vatica pachyphylla* Merr	12	CR	CR	Seasonal, primary dipterocarp forests. Lower elevations	

Nomenclature follows The International Plant Names Index (IPNI 2020).

^a^*DAO* Department Administrative Order, *IUCN* International Union for the Conservation of Nature, *CR* Critically Endangered, *EN* Endangered, *VU* Vulnerable, *LC* Least Concerned, *ND* No Data.

Bioclimatic variables were sourced from CHELSA at 30 arc seconds resolution (estimated to 1 km^2^ at the equator)^58^, which served as the base data for current projections (see Supplementary Table S1). A pairwise Pearson’s correlation analysis was performed on the bioclimatic variable to exclude highly correlated variables (|r|> 0.7)^59,60^; variables bio02, bio10, bio11, bio13 and bio14 were retained. To model the effects of climate change, future projections of those bioclimatic variables from five best Coupled Model Inter-Comparison Project (CMIP_5_) Global Circulation Models (GCMs)^61^ for Southeast Asia^62^: Canadian Earth System Model, second generation (CanESM2); Community Earth System Model, version 1 of Biogeochemistry (CESM1-BGC); Community Earth System Model, version 5.0 of the Community Atmosphere Model (CESM1-CAM5); Centre National de Recherches Météorologiques, Climate Model version 5 (CNRM-CM5); and Model for Interdisciplinary Research On Climate, version 5 (MIROC5), were also sourced from CHELSA and averaged for two representative concentration pathways (RCPs): RCP 4.5 and 8.5 for 2070^58^. The future climate scenarios assumed that global annual greenhouse gas (GHG) emissions would peak by 2040 and subsequently decline substantially for RCP 4.5, or that GHG emissions would continue to rise until 2070 for RCP 8.5, respectively a “best-case” and “worst-case” scenario (RCP 2.6 was not considered as it was considered to be too unrealistic, assuming peak emissions by 2020 and subsequent decline)^63^. Climate change for the Philippines is projected to increase mean temperatures by 0.9–1.9 °C (RCP 4.5) and 1.2–2.3 °C (RCP 8.5) with greater extremes and fluctuations, and increased mean precipitation with greater frequency and intensity of extreme rainfall events^64^.

An additional eight soil variables from SoilGrids at 250 m resolution were included: bulk density (fine earth), clay content mass fraction, coarse fragments volumetric, sand content mass fraction, cation exchange capacity, soil pH in H_2_O, soil pH in KCl, and available soil water capacity^65^. To reduce multicollinearity and potential overfitting^59^, a principal component analysis (PCA) was performed on the eight soil variables^60^. The first five principal components (PCs) were selected as they explained more than 90% of the total variance (93.74%) (see Supplementary Table S2) and aggregated to match the resolution of bioclimatic variables at 30 arc seconds. All processing and analyses of variables were conducted in software R 3.4.1^66^ using the ‘raster’ package^67^.

### Species distribution modelling

MaxEnt is a widely used machine learning software that estimates a species probability distribution through species-climate correspondence using maximum entropy, while bound by the variables and occurrence data provided^15,68^. MaxEnt was selected among other algorithms as it has been shown to be consistently among the top-performing SDMs for presence-only datasets^69,70^, and model transferability^71,72^. While an ensemble of modelling algorithms approach has been suggested to achieve better predictions^73^, the converse has been suggested as well^72,74^. Nevertheless, we chose to use a single well-tuned model that has been calibrated for model transferability while adopting recommended approaches to prevent over-parameterisation and overfitting^71,72,74,75^.

All 26 species were individually modelled using MaxEnt with species-specific background extent, feature class, and regularisation multiplier (for modelling workflow, see Supplementary Fig. S1). For model tuning of both the background extent and MaxEnt parameters (feature class and regularisation multiplier), the performance scores used to select models were obtained by cross-validating models built from partitioned presence and pseudo-absence. To reduce spatial autocorrelation and improve spatial independence between partitions^76^, a ‘block’ partitioning of equal presence points approach (four geographically separated bins based on equal presence points) was used for species with occurrence points greater than 25 (see^77^). The more spatially independent training data for the models would reveal potential overfitting^76,78^, which is important when modelling climate change projections of species’ suitable habitats. A jackknife approach (*k*-fold cross-validation for which *k* = number of observations) was adopted for species with occurrence points less than or equal to 25, an approach that can identify better performing and less overfitted models built with small sample sizes^79,80^.

The optimal background extent was selected by modelling species with different sets of pseudo-absences that were limited by a distance buffer from occurrence points (i.e., varying the background extent used to generate pseudo-absences), using a Michaelis–Menten model to fit distance buffer and model performance, and selecting the lowest distance buffer for which the model’s performance exceeds the theoretical asymptotic performance value^81,82^. The distance buffer ranged sequentially from 20 to 2000 km at increments of 10 km, which totalled to 199 sets of pseudo-absences and 199 models for each species (default MaxEnt settings were used). A fixed 10 km exclusion buffer was also used to prevent points containing both presence and pseudo-absence^83^. Due to the computational intensity of this tuning process, the ‘block’ approach was used for all species regardless of occurrence number and only 1000 pseudo-absences were selected^84^. A model performance criterion of Area Under the Curve (AUC) was used to assess model performance.

For the selection of MaxEnt parameters, an extensive round of species-specific tuning was performed for the model’s feature class and regularisation multiplier. A total of five combinations of the available feature classes (L, LQ, H, LQH, LQPH; Linear, Quadratic, Hinge and Product)^77,85,86^, and 19 regularisation multipliers determined using a geometric progression with a common ratio of 0.75 for eight sequences with a scale factor of 1 based on the default, and a sequential increase of 0.5 from the default 1 to 6.0, was considered. The wider than normal range of regularisation multipliers allowed the evaluations of candidate models with larger values while still accounting for relatively small changes that could also affect model performance^80,87,88^. The five combinations of feature classes and 19 regularisation multipliers generated 95 unique combinations of parameters that were used to build candidate MaxEnt models for each species; 10,000 randomly sampled pseudo-absences limited by the exclusion buffer and background extent determined in the previous tuning was used^82–84,89^.

Candidate models with the lowest Omission Rates (OR) based on the lowest presence threshold (also minimum training presence) were selected^79,90^, assuring against the selection of models overfitted to the calibration data. In cases where more than one candidate model had the lowest OR, the model with the highest AUC was selected as the most optimal^91^. Selected models were then and used to project the species’ geographical suitability in the Philippines for the current and future climate scenarios. While Akaike Information Criterion corrected for small sample size (AICc) has been recommended for the selection of MaxEnt models to prevent overfitting^77,88^, it has been shown to lead to oversimplified models with low predictive performance^46,92^, which was also observed for this study. Thus, OR and AUC with an appropriate cross-validation approach to prevent overfitting and improve model transferability was selected as the criterion for model selection^77–79,87^. The tuning of parameters was conducted in R using the ‘ENMeval’ and ‘SDMtune’ packages^77,93^.

Selected models were evaluated with AUC, OR, and True Skill Statistics (TSS), based on the maximising the sum of sensitivity and specificity (maxSSS) threshold for TSS and OR^94^, i.e., true positive rate and true negative rate. Models of species with AUC < 0.75^91^, TSS < 0.45^95,96^, or OR > 0.25^46,90^, were excluded due to their poor predictive accuracy. Though largely arbitrary^78,97^, it allowed for an assessment of model performance and the exclusion of underperforming models. The final models for each species were then used to project their potential distributions across the Philippines under different climate scenarios. The continuous outputs of habitat suitability were converted into binary maps using the maxSSS threshold^94^.

### *Post-hoc* land cover correction

Considering potential temporal mismatches from using land cover as a predictor variable and the required integration of habitat loss from anthropogenic land use, the land cover was incorporated post-hoc through land cover correction (LCC). This was done by masking areas of unsuitable land cover types; in essence, using land cover to correct the distribution of suitable habitat. To create the masking layer, a static land cover map of the Philippines was sourced from PhilGIS.org and converted to a binary map, with each land cover type reclassified as either ‘suitable’ or ‘unsuitable’ for dipterocarps (see Supplementary Fig. S2). The same unsuitable land cover type was then used to mask out model projections of environmental suitability, for both current and future climate scenarios.

Of the 22 land cover types present in the land cover map, 17 were considered unsuitable for dipterocarps in this study (see Supplementary Table S3). A land cover type was considered unsuitable if it was an anthropogenic land use type (e.g., coconut plantation, quarry, built-up area), while suitable if it was a natural land cover type with intact habitat. This was a reasonable assumption given the focus on tree species, and that anthropogenic land use would entail clearing the land of its forests. Among natural land cover types, only mangrove was considered unsuitable. A conservative approach was adopted for mosaic land cover types, which were designated as unsuitable. Although mosaic landscapes are characterised by forest conversion, degradation, and replacement by non-forest tree cover (e.g., plantations), they may still be ecologically important and potentially suitable for a variety of species^98,99^. The scattered fragments could also potentially be an important accompaniment to larger forests nearby^100^, even though such fragmented habitats would inhibit the reproductive potential of resident individuals^6,7^. Nevertheless, our approach restricted suitable habitat to areas critical for conservation by combining intact habitats, distribution of critical species, and habitat stability under climate change scenarios. Thus, the final distributions from our models (with LCC applied) represented higher conservation priority landscapes, as opposed to a general species distribution that includes areas of potentially less definitive suitability^14,34,40,101^.

### Analysis of outputs

For each species, a total of six suitable habitat distribution scenarios were projected using three climate scenarios (current, RCP 4.5 and RCP 8.5) and two LCC scenarios each (with and without LCC). Changes in suitable habitat for each species were classified as loss, gain, and stable. Loss referred to areas that were originally suitable for the species but were unsuitable after climate change. Gain referred to areas that were initially unsuitable for the species but became suitable after climate change. Stable referred to areas that persisted as suitable habitat after climate change, which have also been referred to as species-specific *in-situ* climatic macrorefugia^39^. Stable areas were stacked to generate and map areas that remained suitable for multiple species.

Changes in suitable habitat for each species were calculated for areas within demarcated protected areas of the Philippines, obtained from the World Database of Protected Areas^102^. This allowed assessing the extent of protection afforded to species—both individually and in aggregate—for current and future climate scenarios. This was particularly important for evaluating stable areas, which should be given a high conservation priority owing to their continued suitability for dipterocarps under climate change.

To further examine the impact of climate change on the distribution of dipterocarps, elevation values for each species projection of suitable habitat—including loss, gain, and stable—was extracted from the GTOPO30 Digital Elevation Model^103^. We calculated the frequency values for each species across elevation bins at 50 m intervals. Raw frequency values were then scaled against the maximum value obtained for the current climate scenario, where the scaled value of 1 would represent the maximum frequency of currently suitable habitats observed across elevation bins (i.e., peak elevation distribution). The scaled values were then aggregated and used to analyse the potential shifts in elevation distribution for dipterocarps. All analyses were conducted in R with the ‘raster’ package^67^.

## Results

Of the 26 species modelled, only 19 were accepted with an average AUC of 0.91 (s.d. 0.05), TSS of 0.69 (s.d. 0.13) and OR of 0.13 (s.d. 0.06) (see Supplementary Table S4). Although occurrence records were low (< 30) for several species, our methods had minimised the potential for model over-parameterisation and overfitting; species with low occurrence records generally had simpler models with larger beta multipliers and a single feature class (see Supplementary Table S4), and had most of their predictors excluded by the lasso penalty in MaxEnt (see Supplementary Table S5)^68,86^.

**Figure 1:**
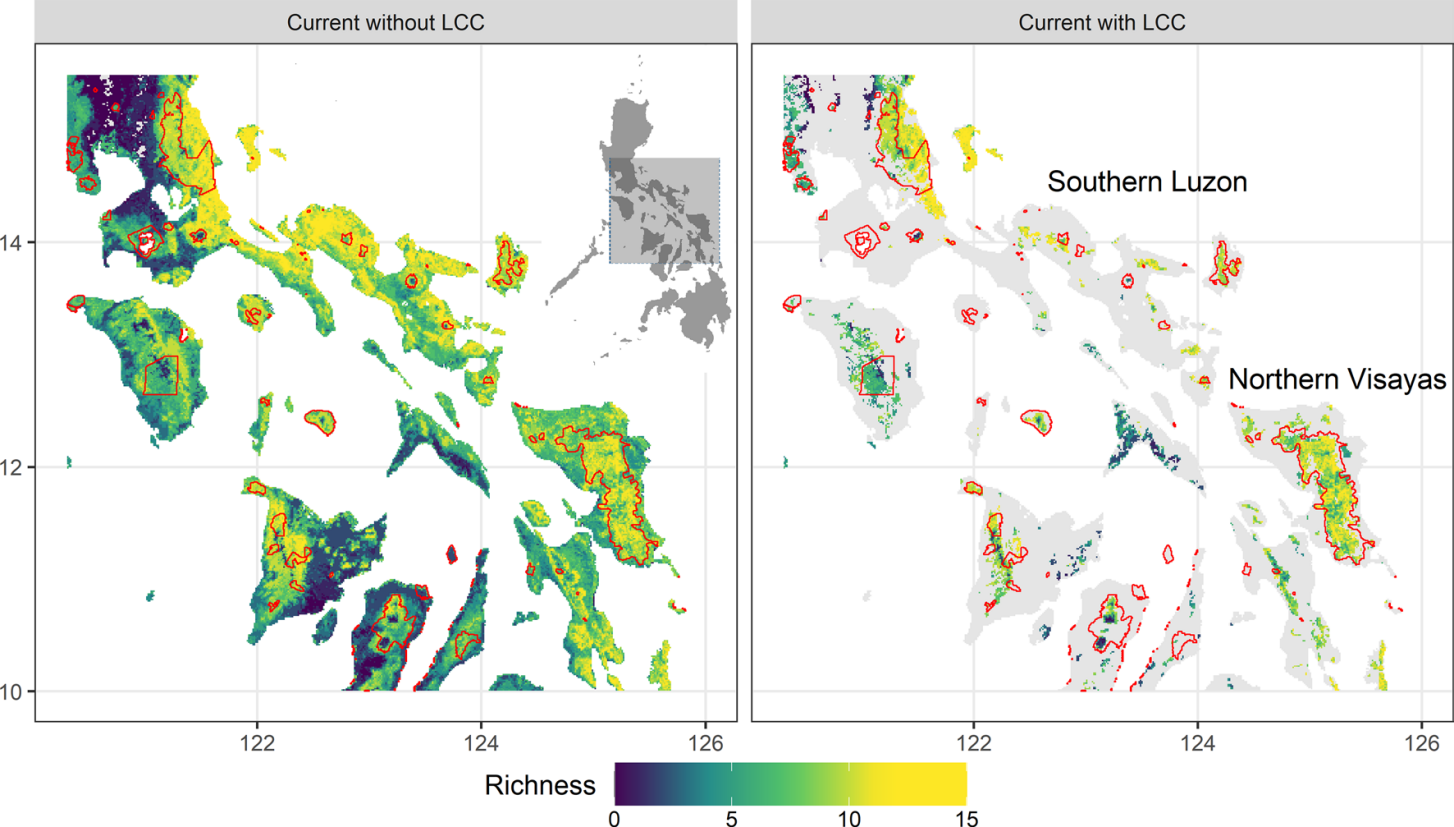
The effects of LCC on the current distribution of suitable habitat for 19 dipterocarps for southern Luzon and northern Visayas, Philippines, which was the region in the Philippines projected to have the largest distribution of species rich suitable habitats. The viridis colours indicate dipterocarp richness—lighter colours indicated higher richness and darker colours indicated lower richness—and protected areas were demarcated in red. The maps were created in R using the raster and ggplot2 package^66,67,121^.

Correcting for land cover resulted in a significant reduction of suitable habitat area across species (Fig. 1). All species experienced a minimum 50% reduction of suitable habitat from LCC, with a median of 66.6% (s.d. 6.4%) at the national level and a median of 36.6% (s.d. 10.2%) within protected areas (Table 2); some of the greatest reductions occurred in southern Luzon and the northern Visayas (Fig. 1). Because of the severe reduction of suitable habitat with the application of LCC, all estimates of shifting potential species distributions under climate change were done using land cover-corrected distributions.

**Table 2:** The total potential distribution to the nearest km^2^ for each species with LCC applied, at the National level and within protected areas (PA), for the current climate scenario.

	Current				Future (RCP 4.5)				Future (RCP 8.5)			
	National level		Within PA		National level		Within PA		National level		Within PA	
	with LCC	Reduced (%)	with LCC	Reduced (%)	Loss (%)	Gain (%)	Loss (%)	Gain (%)	Loss (%)	Gain (%)	Loss (%)	Gain (%)
*D. gracilis*	65,048	**67**	21,452	40	16.3	6.4	6.6	5.8	31.3	10.3	20.0	9.4
*D. grandiflorus*	62,852	**71**	20,137	41	6.5	8.1	1.1	9.1	8.5	12.7	1.6	13.8
*D. hasseltii*	56,088	**71**	19,633	40	3.3	0.0	2.4	0.0	1.8	0.4	1.0	0.3
*D. kunstleri*	8097	**74**	559	**68**	10.0	13.2	4.4	62.9	13.2	30.6	9.4	157.3
*D. validus*	10,599	**81**	2424	48	0.0	110.1	0.0	116.3	0.0	193.9	0.0	212.4
*H. acuminata*	22,475	**63**	7904	34	35.2	43.9	36.2	43.6	**56.0**	62.7	**58.0**	64.1
*H. malibato*	28,181	**63**	10,459	32	14.1	33.0	11.2	27.5	19.2	47.5	11.7	43.1
*H. plagata*	63,728	**63**	21,216	37	2.0	1.0	1.2	0.6	3.0	1.5	2.1	0.8
*S. almon*	58,762	**70**	17,747	37	6.7	2.7	3.8	4.4	7.9	5.2	2.3	8.6
*S. assamica*	33,378	**63**	12,428	36	34.6	8.6	39.2	6.0	**50.2**	10.1	**54.4**	7.2
*S. astylosa*	3921	**61**	1018	28	**68.1**	32.8	**63.2**	32.9	**87.5**	35.5	**86.3**	46.6
*S. contorta*	42,472	**67**	17,674	36	5.2	9.2	2.4	9.0	8.3	14.5	5.3	13.0
*S. guiso*	56,517	**67**	18,130	37	19.1	14.8	13.3	20.3	24.9	17.5	19.8	23.5
*S. hopeifolia*	19,950	**62**	4734	31	**55.9**	17.7	**50.8**	30.0	**71.8**	19.2	**69.4**	30.8
*S. ovata*	8322	**59**	1954	26	39.9	50.7	19.8	92.5	**56.9**	53.0	37.3	90.5
*S. palosapis*	19,441	**54**	7802	25	38.3	22.7	39.8	22.8	**57.1**	37.5	**59.1**	39.2
*S. polysperma*	44,275	**60**	16,496	30	15.1	15.5	15.7	11.2	27.0	20.8	27.6	15.2
*S. virescens*	5185	**56**	244	**54**	45.4	42.7	**88.6**	78.0	**69.9**	64.4	**86.2**	128.7
*V. pachyphylla*	5663	**68**	949	36	**50.8**	36.7	49.5	88.8	**63.2**	30.8	**61.6**	89.1
Median		63.5		36.3	16.3	15.5	13.3	22.8	27.0	20.8	20.0	30.8

Reduced, indicates the percentage of the species distribution that was removed due to LCC. Future projections show as a percentage of the current distribution (with LCC), suitable area loss, refugia, or gain for both the National level and Within PA. Projections were done for RCP 4.5 and RCP 8.5 for 2070. Percentages in bold indicate a greater than 50% loss of suitable habitat.

**Figure 2:**
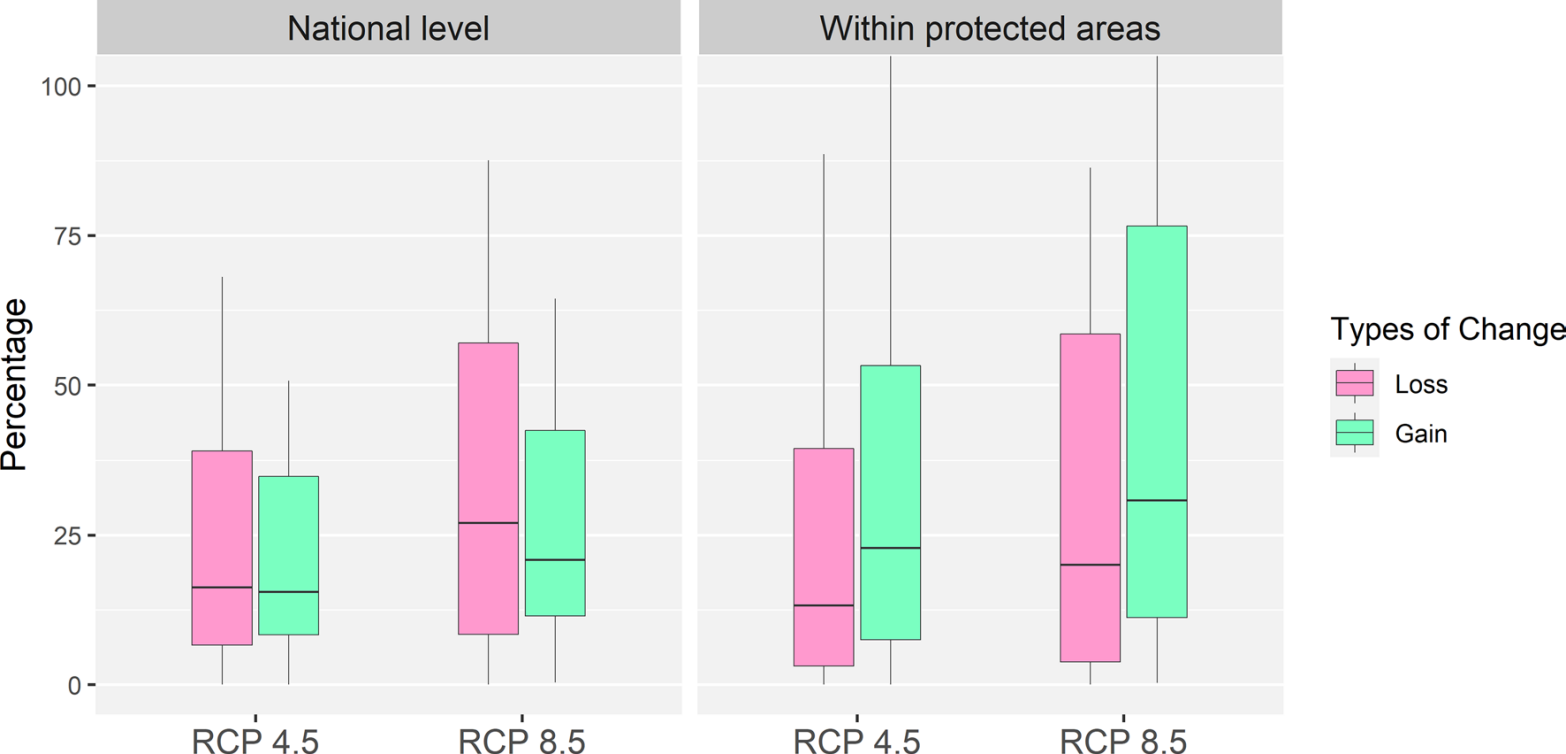
Percentage of suitable habitat loss (red) and gain (green) from climate change, relative to current predictions with LCC, for 19 dipterocarp species in the Philippines under two future climate scenarios (RCP 4.5 and RCP 8.5 for 2070), at the national level and within protected areas. The figure was created in R using the ggplot2 package^66,121^.

At the national level, climate-induced losses were projected at a median of 16.3% _RCP 4.5_ (IQR 6.6%–39.1%) and 27.0% _RCP 8.5_ (IQR 8.4%–57.0%), with gains of 15.5% _RCP 4.5_ (IQR 8.3%–34.8%) and 20.8% _RCP 8.5_ (IQR 11.5%–42.5%) (Fig. 2). Species-specific responses varied substantially, with losses ranging from 0% (*D. validus*) to 87.5% (*S. astylosa*), and gains of 0.4% (*S. almon*) to 193.9% (*D. validus*) for RCP 8.5 (Table 2). Losses and gains for RCP 4.5 were significantly less than RCP 8.5 (pairwise T-test, *n* = 19, *P*_Loss_ < 0.001; *P*_Gain_ = 0.015), with a mean difference of 10.1% for loss and 10.4% for gain.

Changes in suitable habitat within protected areas showed similar trends: median losses of 13.3% _RCP 4.5_ (IQR 3.1%–39.5%) and 20.0% _RCP 8.5_ (IQR 3.8%–58.6%), and gains of 22.8% _RCP 4.5_ (IQR 7.5%–53.2%), and 30.8% _RCP 8.5_ (IQR 11.2%–76.6%) (Fig. 2). Losses and gains within protected areas for RCP 4.5 were also significantly less than RCP 8.5 (pairwise T-test, *n* = 19, *P*_Loss_ < 0.001 and *P*_Gain_ = 0.010), with a mean difference of 8.6% for loss and 17.5% for gain. While losses within protected areas were not significantly different from those at the national level (pairwise T-test, *n* = 19, *P*_RCP 4.5_ = 0.741; *P*_RCP 8.5_ = 0.162), gains within protected areas were significantly greater (pairwise T-test, *n* = 19, *P*_RCP 4.5_ = 0.033; *P*_RCP 8.5_ = 0.038).

**Figure 3:**
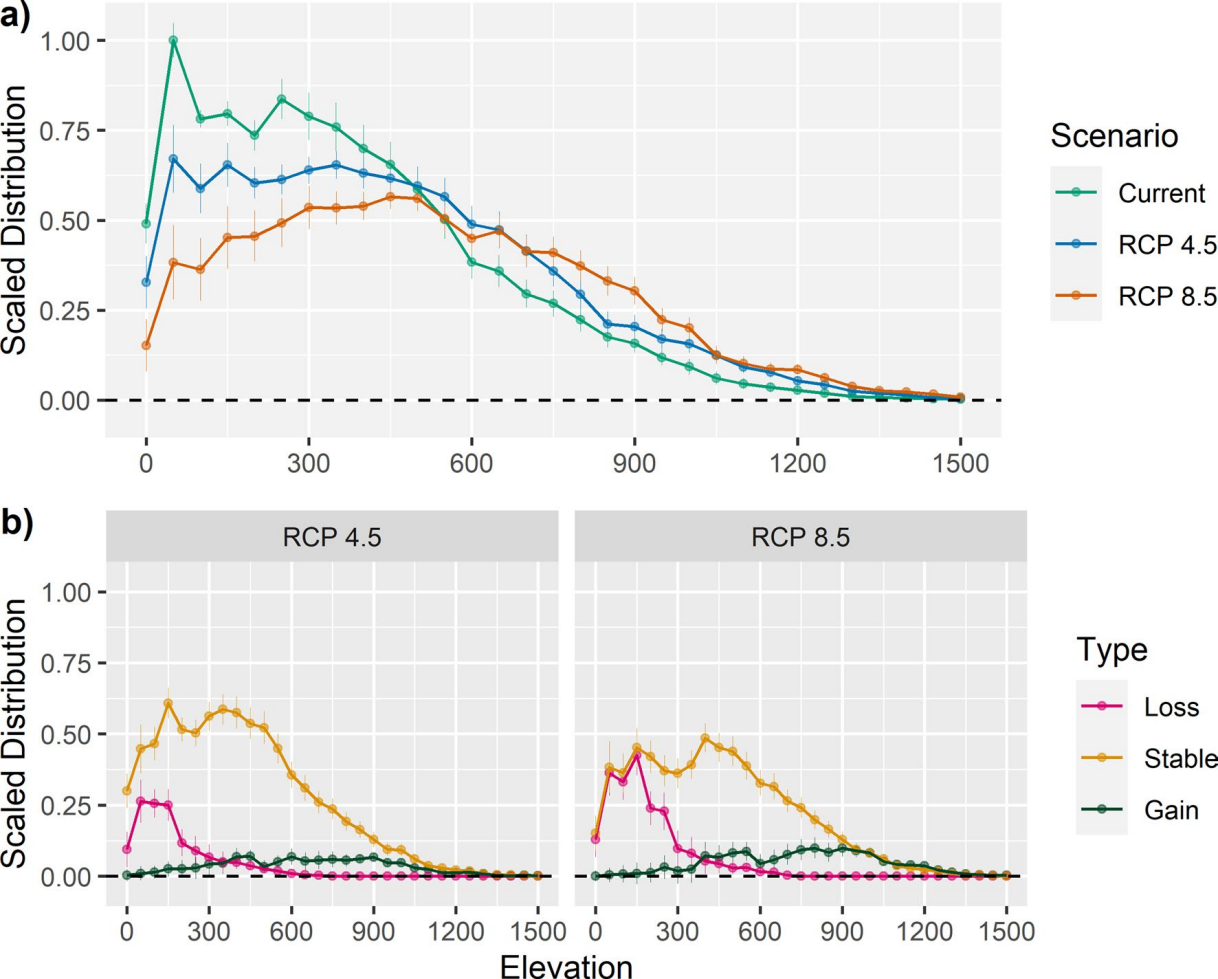
(**a**) The averaged scaled distribution of dipterocarps—with LCC—along an elevation gradient for the current and two future climate scenarios (RCP 4.5 and RCP 8.5 for 2070); (**b**) and changes in those distributions from current to future as loss, stable and gain, for each of the two future climate scenarios in the Philippines. Scaled distributions indicate the distribution of suitable habitat—or for any of the three potential changes in suitable habitat—across elevation bins of 50 m intervals scaled against the maximum obtained for any bin under the current climate scenario, where 1 represented the peak distribution among bins. The figure was created in R using the ggplot2 package^66,121^.

Under projections of climate change, species distributions of suitable habitat across the elevation gradient experienced shifts. The scaled elevation distributions averaged across species decreased at elevations below 400 m asl and increased at 600–900 m asl from current to both future climate scenarios (Fig. 3a). These changes corresponded well to gross losses and gains, indicating a general upward trend in species elevation distribution, most evident under RCP 8.5 (Fig. 3b). The dip in stable areas at 300 m asl—which resulted in twin peaks—is attributable to high losses at 100–300 m asl for a subset of about eight dipterocarp species (see Supplementary Fig. S3 for species-specific shifts).

**Figure 4:**
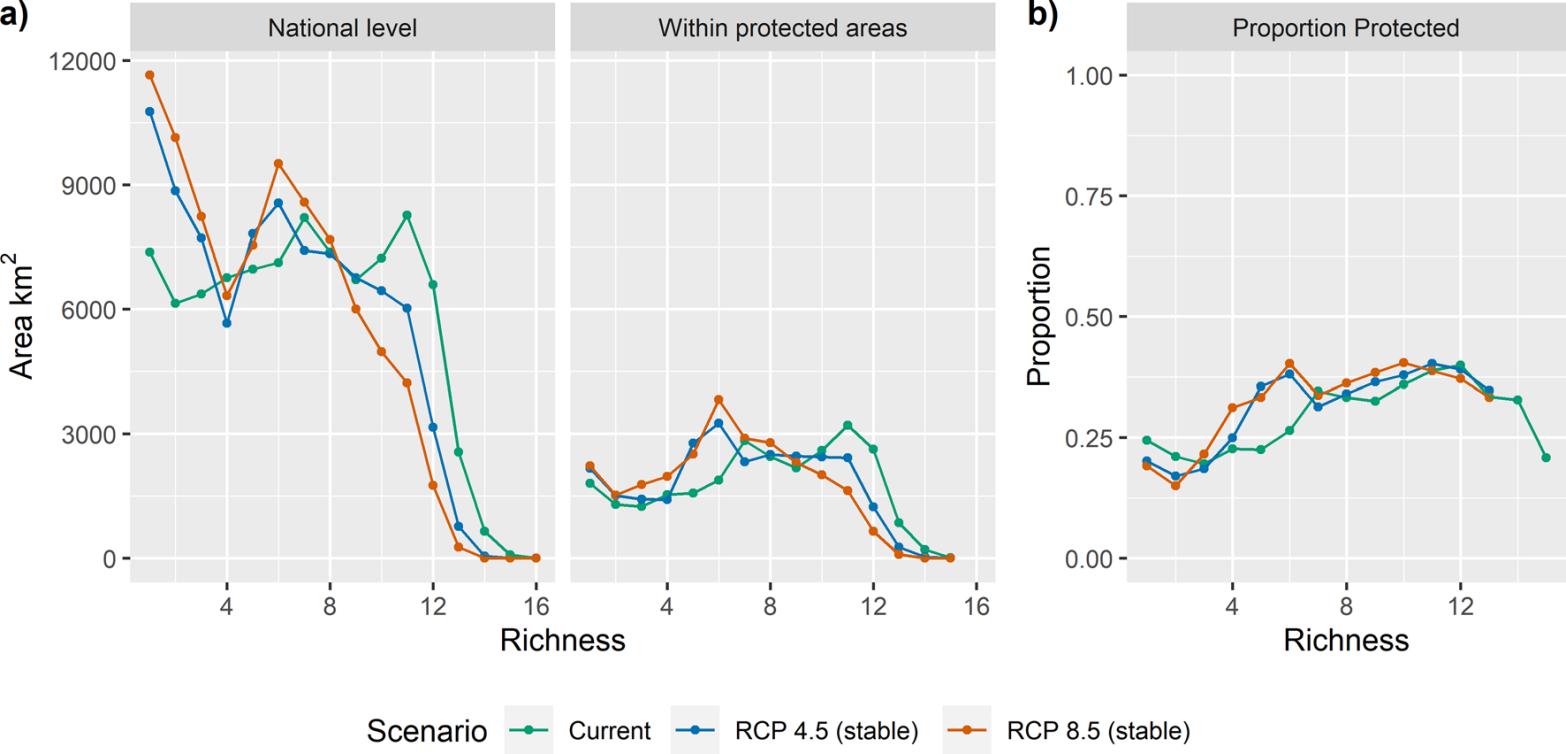
(**a**) The total area by species richness for the current and two future climate scenarios (RCP 4.5 and RCP 8.5 for 2070)—only stable areas were calculated for future climate scenarios—at the national level and within protected areas only for dipterocarp species in the Philippines. (**b**) The proportion of the area by species richness at the national level that exists within protected areas. Proportion data was excluded if the area was less than 50 km^2^ (seen as absent points), as it may lead to arbitrarily high proportion protected values. The figure was created in R using the ggplot2 package^66,121^.

At the national level for the current climate scenario, there was a relatively even distribution of areas outside national parks that contained fewer than 12 dipterocarp species (6000–8000 km^2^) (Fig. 4a). In contrast, species-rich areas (> 12 species) comprised a total of ca. 3000 km^2^ combined (Fig. 4a). Under both RCP scenarios, areas with low species richness (1–8) increased while areas with higher richness decreased, suggesting an erosion of species richness under climate change.

**Figure 5:**
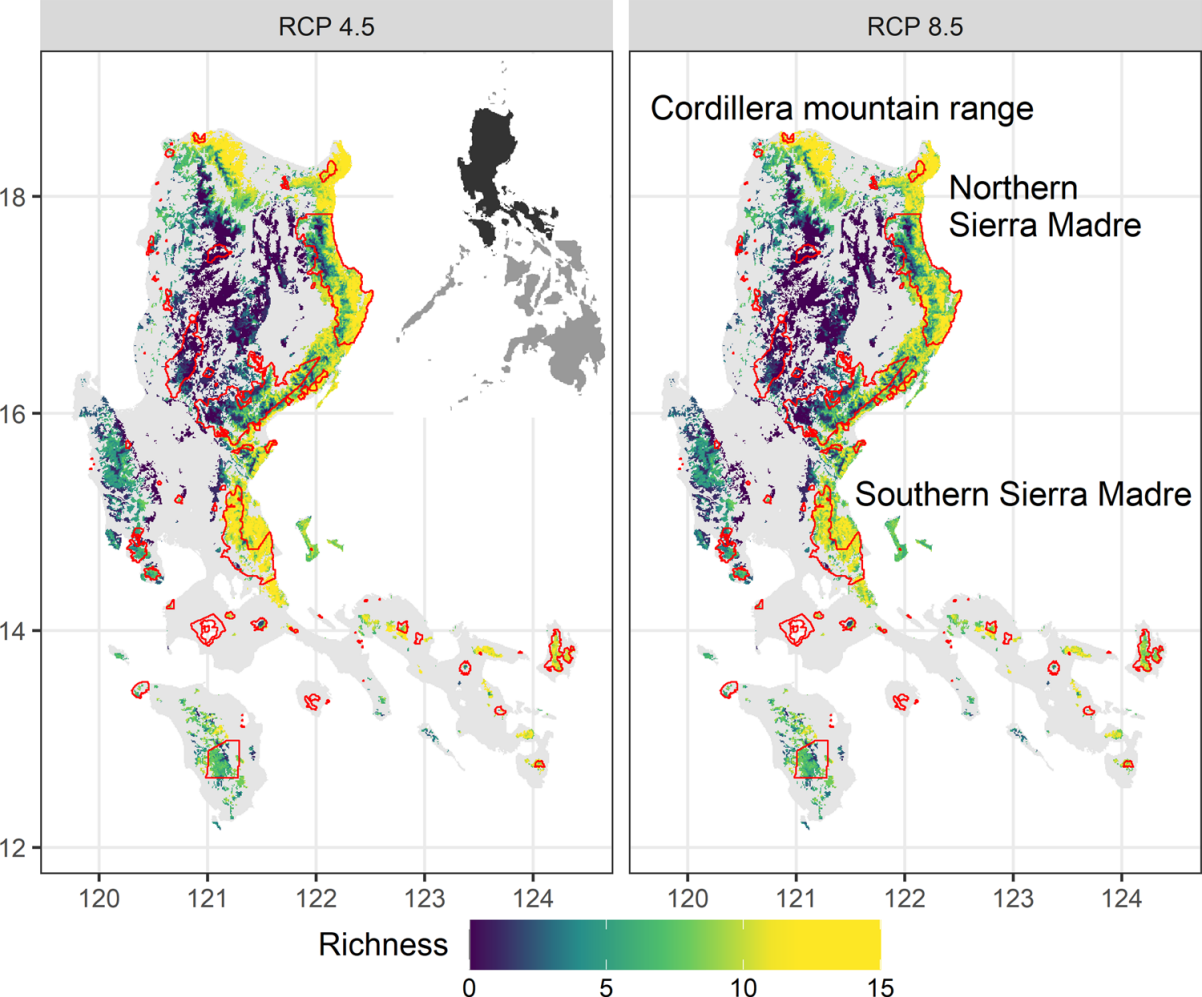
The stacked distribution of stable areas for 19 dipterocarp species in Greater Luzon, Philippines under climate scenarios, RCP 4.5 and RCP 8.5 for 2070; viridis colours indicate dipterocarp richness—lighter colours indicated higher richness and darker colours indicated lower richness—and protected areas were demarcated in red. Areas of especially high species richness but were not currently protected include lowland regions of the northern Sierra Madre, southern Sierra Madre and Cordillera mountain ranges. The maps were created in R using the raster and ggplot2 package^66,67,121^.

Protected areas, which comprised a small proportion of total area, mirrored the trends observed at the national level for current and both future climate scenarios (Fig. 4b). The proportion of area covered by protected areas increased with richness (Fig. 4b), in line with the mandate of protected areas to focus on species-rich regions. Importantly, areas designated as stable under climate change were poorly covered by the existing protected area system (Fig. 5). This was particularly notable for areas with the highest species richness. Areas of high dipterocarp richness that were left unprotected included the lowlands of northern and southern Sierra Madre, and Cordillera mountain ranges for the Greater Luzon region (Fig. 5).

## Discussion

### Post-hoc land cover correction of SDMs

Incorporating land cover into species distribution models in dynamic geographic regions such as Southeast Asia presents a considerable challenge for model accuracy, because of temporal mismatches between (historical) occurrence records and (contemporary) land cover data. Indeed, previous research investigating changes in suitable habitat under climate change typically did not include land cover^11,104^ or included it as a variable in the model itself^12,105,106^. Here, we incorporated land cover post-hoc, which allowed us to leverage the maximum amount of occurrence-environment data without incorporating errors generated from those temporal mismatches ^46,47^. As such, our approach directly accounted for the spatial constraints imposed by anthropogenic land use when assessing the impacts of climate change^36,41^. Accounting for anthropogenic land use is particularly important for the Philippines as dipterocarps are typically lowland species^25,26^, many of which are threatened with habitat loss as a major driver for their decline^51,52^, and the tropical lowlands have been the hardest hit by deforestation^22,23^. Indeed, the Philippines has only 3% of its primary forest remaining as a result of forest conversions to anthropogenic land use^22,23^.

Incorporating LCC, therefore, imposed significant and necessary spatial constraints on the extent of both current and future species distributions; this was a conservative approach that prevented the overestimation of species future distribution, which would have resulted in an underestimation of a species conservation priority^36,42,107^. For example, although *D. validus* and *H. plagata* were projected under either climate change scenario to experience almost no loss of suitable habitat, both had their potential distributions substantially reduced with LCC, which allowed for a more thorough assessment of future threats to species distributions. Constraining species distributions based on land cover suitability has important implications for conservation interventions, as improved spatial assessments will facilitate geographically targeted allocation of resources towards mitigating biodiversity loss^13,14,37,108^.

The post-hoc LCC approach has broad applicability when projecting the effects of climate change on species in regions that have experienced rapid recent land cover change, and for regions that are poorly surveyed—conditions that describe much of the global tropics^16–18,20^. Importantly, the corrections applied in this study were based on a static contemporary land cover map. For the Philippines, this is unlikely to be a problem because the vast majority of quality forest has already been lost, while forest loss in the majority of provinces has been less than 6% from 2000–2014 (J. D. De Alban, unpublished data). However, in regions where deforestation rates are high or expected to increase going forward, such as Cambodia^109^ or Indonesia^16^, it may be more appropriate to incorporate projections of future land cover^36,42^.

### Climate-induced shifts and climate macrorefugia for dipterocarpaceae

Our results revealed a high degree of variability in dipterocarp responses to climate change. While some species were projected to remain relatively unaffected by climate change, others were projected to experience a substantial loss of suitable habitat, especially along lower elevations. Suitable habitat loss for a species indicates, at a minimum, an increase of environmental pressure favouring mortality over recruitment in those locales, which may ultimately manifest in local extirpations or functional extinctions^110,111^. Consequently, this could trigger bottom-up effects on plant-animal mutualistic networks, such as extinction cascades, which have been predicted to arise from the extinction of other plant species^30–33^. The differential response to climate change across closely associated dipterocarp species further suggests the potential for future disaggregation of tree communities, which will affect the structure and function of the forests they form^27,29,31^. This is especially true for dipterocarps, considering their dominance and structural importance across forests in SEA^24,28,29^, which further reinforces the importance of dipterocarp persistence, and conservation efforts to ensure it^27–29,35^.

An important outcome of this study was the projections of suitable habitat that remained so under climate change—identified as stable in this study. These areas represent species-specific in-situ climatic macrorefugia that can potentially secure the persistence of species under climate change^34,39,40^. The availability of climatic macrorefugia is especially vital for species with dispersal limitations and long maturation time, which would hinder a species’ ability to rapidly migrate following projected shifts in suitable habitat^39^. This applies to dipterocarps, as they generally exhibit both traits^24,25,112,113^. The loss of these areas would mean the loss of source populations, potentially limiting the recovery and survival of species following climate change^27,34,35,39^. With the projected low availability of such refugia for several of the species studied, climatic macrorefugia represent areas of exceptional conservation importance.

### Protected areas to safeguard climate macrorefugia

The goal of protected areas is to safeguard species both now and for future generations, the latter of which is gaining relevance given the large projected shifts in climatic conditions for protected areas globally^64,114^. Our findings highlight a critical gap in the Philippines protected area system in that it covers a relatively small proportion of suitable habitat for dipterocarps currently, and a similarly low proportion of species-rich climate macrorefugia under both RCP scenarios. Given the importance of dipterocarps in maintaining the structure and function of forest in SEA, a persuasive case can be made to integrate dipterocarp distributions into protected area network designs^13,14,34,101^.

Most existing protected areas have been designed based on the current distribution of species and richness^19,106,115^, and progressive planning will need to incorporate species range shifts under climate change^13,116^. Our study affirms the importance of integrating SDMs and species refugia into long-term planning of protected areas^13,14,34,39,40,101,116–118^. Here we highlight three applications of this research for incorporating SDMs into protected area planning. First, individual or stacked maps of suitable habitat loss would reveal protected areas most heavily affected by climate change, and consequently, most at risk of underperformance^101,108,114^. Second, mapping areas of continued habitat suitability for multiple species will allow the identification of species-rich climatic macrorefugia inside and outside protected areas, therefore prioritizing conservation effort for dipterocarps^34,39,40^. For example, the lowlands of northern and southern Sierra Madre, and Cordillera mountain ranges were identified as unprotected species-rich areas for Greater Luzon. When applied across the entire country, a larger set of priority landscapes would emerge to support site-specific, long-term conservation planning for the protected area system, ensuring the increased coverage of critically important species-rich climatic macrorefugia. Third, distribution maps of suitable habitat gain highlight potentially biodiverse regions in the future, which could be integrated into protected areas over the long-term^13,14,114^, notwithstanding the underlying assumption of full dispersal, which is largely unrealistic for dipterocarps owing to dispersal limitations^112,113,119^.

Finally, these SDM outputs could contribute to long-term spatially-explicit conservation planning through platforms such as Marxan, which demarcates protected areas based on set conservation targets at a minimal total cost^120^. Alongside suitable habitat projections of other key taxa and the inclusion of various cost metrics, our results could help indicate cost-effective areas for protected area expansion in the Philippines^40,120^. Moreover, with increasing commitments to restoring forest cover in SEA, suitable habitat projections before LCC would provide crucial information on locations for which dipterocarp reforestation could be done successfully^13,14,17,27,35^. Thus, the stacked maps of loss, stable, and gain can directly aid in informing more effective planning of conservation priorities and reforestation projections, better preparing protected areas for the challenges of protecting species under climate change^40,117,118^.

## Supplementary Information

Supplementary Information.

## Data Availability

Occurrence data of threatened tree species are accessible from Ramos et al.^21^ and GBIF^49^. Bioclimatic and soil variables are publicly accessible from CHELSA-climate.org and SoilGrids.org, respectively. The raw data of the results produced from this study can be found in the Supplementary material.
