# Supplementary material for: Effects of climate change and land cover on the distributions of a critical tree family in the Philippines
Source: Sci Rep. 2021 Jan 11;11:276. doi: 10.1038/s41598-020-79491-9 (PMC7801684; doi:10.1038/s41598-020-79491-9)
Supplement: Supplementary file 1 — Supplementary Information. [file 41598_2020_79491_MOESM1_ESM.docx]

**Supplementary Material**

**Title:** Effects of climate change and land cover on the distribution of a critical tree family in the Philippines

**List of Authors:** Sean E. H. Pang* ^1^, Jose Don T. De Alban ^1^, Edward L. Webb* ^1^

**Institutional affiliation:** ^1^ Department of Biological Sciences, National University of Singapore, 117543 Singapore

**Contact Information:** SEHP: +65 8223 7223 (s.pang@u.nus.edu); ELW: +65 6516 4184 ([ted.webb@nus.edu.sg](mailto:ted.webb@nus.edu.sg))

**Contents**

Table S1: List of predictors used for species distribution modelling

Table S2: Summary statistics and loadings of the PCA for bioclimatic variables

Table S3: Land cover types and their suitability for dipterocarps in the Philippines

Table S4: Final model parameters and performance

Table S5: Final model variable permutation importance

Table S6: Absolute area of different types of changes to suitable habitat for 19 dipterocarps across different scenarios

Table S7: MESS analysis for each climate scenario and for each species

Figure S1: Workflow for the entire modelling process

Figure S2: Map of land cover types and their suitability for dipterocarps in the Philippines

Figure S3: Species-specific scaled elevation distribution of dipterocarps in the Philippines

Figure S4: Species-specific effects of land cover and climate change simultaneously for dipterocarps in the Philippines

Figure S5: Stacked current and future suitable habitat distribution of dipterocarps in the Philippines

Figure S6: Stacked suitable habitat refugia of dipterocarps in the Philippines under climate change

Figure S7: Stacked suitable habitat loss of dipterocarps in the Philippines under climate change

Figure S8: Stacked suitable habitat gain of dipterocarps in the Philippines under climate change

**Supplementary**

Reductions from the application of LCC cascaded into large revisions of estimates of climate-induced losses and gains of suitable habitat. For example, without LCC, the median climate-induced habitat loss under RCP 8.5 (N=20 species) was 37.7% of the current extent (without LCC); when calculated after LCC the median loss was reduced to 9.2% of the current extent (without LCC). Similarly, area of refugia (RCP 8.5) was reduced from a median of 62.3% to 21.8%, and habitat gain was reduced from a median of 15.4% to 8.1% (Figure S3).

**Table S1.** A list of bioclimatic and biophysical variables with their full names, which were used for the modelling process of MaxEnt species distribution modelling

| Name | Full name | Source |
| --- | --- | --- |
| Bio 2 | Mean Diurnal Range | CHELSA |
| Bio 10 | Mean Temperature of Warmest Quarter | CHELSA |
| Bio 11 | Mean Temperature of Coldest Quarter | CHELSA |
| Bio 13 | Precipitation of Wettest Month | CHELSA |
| Bio 14 | Precipitation of Driest Month | CHELSA |
| AWC | Available Soil Water Capacity | SoilGrids |
| BLDFIE | Bulk density (fine earth) | SoilGrids |
| CECSOL | Cation Exchange Capacity | SoilGrids |
| CLYPPT | Clay content mass fraction | SoilGrids |
| CRFVOL | Coarse Fragments Volumetric | SoilGrids |
| PHIHOX | Soil pH in H_2_O | SoilGrids |
| PHIKCL | Soil pH in KCL | SoilGrids |
| SNDPPT | Sand Content Mass Fraction | SoilGrids |

**Table S2.** Summary statistics and principal component (PC) loadings for the principal component analysis of the 8 soil variables. Only the first 5 PC axis were selected as predictors, which accounted for 90% of all variance present

|  | **PC Summary Statistics** | | | | | | | | | | | | | | |
| --- | --- | --- | --- | --- | --- | --- | --- | --- | --- | --- | --- | --- | --- | --- | --- |
|  | **PC1** |  | **PC2** |  | **PC3** |  | **PC4** |  | **PC5** |  | **PC6** |  | **PC7** |  | **PC8** |
| **Standard deviation** | 1.70 |  | 1.32 |  | 1.16 |  | 0.98 |  | 0.75 |  | 0.59 |  | 0.35 |  | 0.19 |
| **Proportion of Variance** | 0.36 |  | 0.22 |  | 0.17 |  | 0.12 |  | 0.07 |  | 0.04 |  | 0.02 |  | 0.00 |
| **Cumulative Proportion** | 0.36 |  | 0.58 |  | 0.75 |  | 0.87 |  | 0.94 |  | 0.98 |  | 1.00 |  | 1.00 |
|  | **PC loadings for each variable** | | | | | | | | | | | | | | |
|  | **PC1** |  | **PC2** |  | **PC3** |  | **PC4** |  | **PC5** |  | **PC6** |  | **PC7** |  | **PC8** |
| **AWC** | -0.417 |  | 0.143 |  | -0.283 |  | -0.589 |  | -0.116 |  | 0.055 |  | 0.061 |  | -0.598 |
| **BLDFIE** | 0.486 |  | 0.131 |  | 0.410 |  | 0.153 |  | 0.139 |  | -0.179 |  | -0.089 |  | -0.704 |
| **CECSOL** | 0.009 |  | -0.224 |  | -0.602 |  | 0.621 |  | -0.313 |  | 0.010 |  | 0.042 |  | -0.320 |
| **CLYPPT** | -0.065 |  | -0.673 |  | 0.240 |  | -0.023 |  | 0.215 |  | 0.421 |  | 0.485 |  | -0.160 |
| **CRFVOL** | 0.294 |  | 0.084 |  | -0.538 |  | -0.096 |  | 0.777 |  | 0.045 |  | 0.042 |  | 0.021 |
| **PHIHOX** | 0.503 |  | 0.025 |  | -0.153 |  | -0.299 |  | -0.355 |  | -0.309 |  | 0.630 |  | 0.127 |
| **PHIKCL** | 0.490 |  | -0.064 |  | -0.112 |  | -0.260 |  | -0.305 |  | 0.650 |  | -0.399 |  | 0.030 |
| **SNDPPT** | -0.077 |  | 0.669 |  | 0.084 |  | 0.279 |  | -0.005 |  | 0.518 |  | 0.440 |  | -0.007 |

**Table S3.** Initial land cover types for the Philippines with their designated suitability towards dipterocarps, the supporting reason for such a designation, and the proportion of the entire landscape consisting of that land cover type. (See Figure S1 for a visual representation)

| **Land cover type** | **Suitability** | **Reason** | **Proportion** |
| --- | --- | --- | --- |
| Closed canopy, mature tree covering >50 percent | Yes | Intact habitat | 0.081 |
| Grassland, grass covering >70 percent | Yes | Intact habitat | 0.061 |
| Mossy forest | Yes | Intact habitat | 0.008 |
| Open canopy, mature trees covering <50 percent | Yes | Intact habitat | 0.142 |
| Unclassified | Yes | Intact habitat | 0.018 |
| Arable land, crops mainly cereals and suger | No | Anthropogenic land use | 0.149 |
| Built-up Areas | No | Anthropogenic land use | 0.005 |
| Coconut plantations | No | Anthropogenic land use | 0.039 |
| Crop land mixed with coconut plantation | No | Anthropogenic land use | 0.122 |
| Crop land mixed with other plantation | No | Anthropogenic land use | 0.013 |
| Cultivated Area mixed with brushland/grassland | No | Anthropogenic land use | 0.331 |
| Eroded area | No | Anthropogenic land use | 0.000 |
| Fishponds derived from mangrove | No | Aquatic | 0.007 |
| Lake | No | Aquatic | 0.007 |
| Mangrove vegetation | No | High salinity | 0.006 |
| Marshy area and swamp | No | Water logged | 0.003 |
| Other barren land | No | Anthropogenic land use | 0.000 |
| Other fishponds | No | Aquatic | 0.000 |
| Other plantations | No | Anthropogenic land use | 0.003 |
| Quarry | No | Anthropogenic land use | 0.000 |
| Riverbeds | No | Aquatic | 0.003 |
| Siltation pattern in lake | No | Aquatic | 0.001 |

**Table S4.** The model performance of the final 19 Dipterocarpaceae species, evaluated using AUC, TSS and POD (1- OR), and their final species-specific parameters (background extent, feature class, and beta)

| **Species** | **Background Extent Buffer (km^2^)** | **Feature Class** | **Beta** | **AUC** | **TSS** | **POD** |
| --- | --- | --- | --- | --- | --- | --- |
| *D. gracilis* | 210 | L | 6.0 | 0.84 | 0.55 | 0.82 |
| *D. grandiflorus* | 330 | LQ | 4.0 | 0.88 | 0.61 | 0.89 |
| *D. hasseltii* | 610 | L | 2.5 | 0.84 | 0.54 | 0.82 |
| *D. kunstleri* | 1010 | LQPH | 3.5 | 0.85 | 0.56 | 0.75 |
| *D. validus* | 1120 | H | 5.0 | 0.94 | 0.71 | 0.81 |
| *H. acuminata* | 820 | L | 4.0 | 0.95 | 0.85 | 0.97 |
| *H. malibato* | 960 | L | 0.42 | 0.96 | 0.83 | 0.94 |
| *H. plagata* | 470 | LQ | 3.0 | 0.85 | 0.61 | 0.90 |
| *S. almon* | 920 | LQH | 2.0 | 0.84 | 0.51 | 0.82 |
| *S. assamica* | 470 | H | 1.5 | 0.95 | 0.77 | 0.90 |
| *S. astylosa* | 450 | L | 0.42 | 0.99 | 0.97 | 1.00 |
| *S. contorta* | 800 | L | 5.5 | 0.90 | 0.67 | 0.87 |
| *S. guiso* | 400 | L | 6.0 | 0.87 | 0.61 | 0.78 |
| *S. hopeifolia* | 1070 | L | 6.0 | 0.87 | 0.58 | 0.81 |
| *S. ovata* | 890 | H | 4.0 | 0.95 | 0.77 | 0.90 |
| *S. palosapis* | 720 | H | 3.5 | 0.97 | 0.86 | 0.91 |
| *S. polysperma* | 750 | LQH | 5.5 | 0.91 | 0.70 | 0.90 |
| *S. virescens* | 1090 | L | 0.42 | 0.92 | 0.73 | 0.88 |
| *V. pachyphylla* | 140 | H | 3.0 | 0.99 | 0.84 | 0.92 |

**Table S5.** The permutation importance of variables—as percentages—for the final models, for each of the 19 Dipterocarpaceae species modelled. Variable permutation importance above 10% were bolded

| Species | Bio 02 | Bio 10 | Bio 11 | Bio 13 | Bio 14 | PC1 | PC2 | PC3 | PC4 | PC5 |
| --- | --- | --- | --- | --- | --- | --- | --- | --- | --- | --- |
| *D. gracilis* | **43.9** | 8.5 | **11.3** | 0.5 | 8.0 | **17.5** | 0.7 | 3.7 | 3.0 | 3.0 |
| *D. grandiflorus* | **24.4** | 0.0 | **32.4** | 1.8 | 0.1 | **20.8** | 1.3 | **15.0** | 4.0 | 0.1 |
| *D. hasseltii* | **80.2** | 1.5 | **15.0** | 0.4 | 0.0 | 0.0 | 1.3 | 1.0 | 0.0 | 0.5 |
| *D. kunstleri* | 0.0 | 0.0 | 7.1 | **23.5** | **47.0** | 0.0 | **20.7** | 1.7 | 0.0 | 0.0 |
| *D. validus* | 6.2 | **28.7** | 9.5 | 2.9 | **33.6** | **11.7** | 1.5 | 5.0 | 0.1 | 0.8 |
| *H. acuminata* | 0.7 | **15.3** | **53.6** | 5.7 | 4.2 | **17.7** | 0.5 | 2.2 | 0.1 | 0.0 |
| *H. malibato* | 1.6 | 1.3 | **31.1** | 5.8 | 4.1 | **36.6** | 2.7 | **13.6** | 2.4 | 0.9 |
| *H. plagata* | 0.0 | 0.0 | 0.0 | 0.0 | **75.9** | 0.0 | 0.0 | 9.1 | 0.0 | **15.0** |
| *S. almon* | 4.9 | **11.1** | 0.5 | 0.6 | **25.8** | **35.8** | 2.2 | **19.0** | 0.0 | 0.0 |
| *S. assamica* | **18.0** | 1.5 | **10.3** | 6.9 | **12.7** | **13.2** | 6.9 | **15.3** | **13.7** | 1.6 |
| *S. astylosa* | 4.0 | 1.7 | **40.4** | 2.5 | **13.9** | **23.3** | 1.6 | 2.3 | 6.8 | 3.6 |
| *S. contorta* | 5.6 | **12.2** | **25.7** | 3.4 | 8.2 | **20.3** | 6.1 | **14.6** | 2.7 | 1.2 |
| *S. guiso* | **38.6** | 1.0 | **18.3** | 3.7 | 6.9 | **11.8** | 6.3 | **11.2** | 2.0 | 0.2 |
| *S. hopeifolia* | **10.0** | **15.2** | 9.3 | 0.4 | **32.4** | **20.8** | 1.5 | 8.1 | 2.4 | 0.0 |
| *S. ovata* | **12.8** | **16.0** | 6.0 | 0.0 | **44.2** | 4.7 | 5.4 | 7.7 | 1.3 | 2.0 |
| *S. palosapis* | **13.8** | 9.2 | **43.5** | 0.0 | 3.2 | 4.8 | 0.0 | **25.4** | 0.0 | 0.2 |
| *S. polysperma* | 0.1 | 0.9 | **30.5** | **15.7** | 0.9 | **37.0** | 0.5 | **13.5** | 0.3 | 0.6 |
| *S. virescens* | **16.4** | 6.2 | 4.5 | 1.4 | **36.7** | **19.1** | 1.7 | 9.7 | 1.3 | 3.0 |
| *V. pachyphylla* | **13.5** | **15.0** | **10.0** | **11.6** | **39.5** | 3.2 | 3.3 | 1.2 | 0.0 | 2.8 |

**Table S6.** The absolute area in km^2^ of suitable habitat for each climatic scenario (RCP 4.5 and RCP 8.5), without or with land cover correction (LCC), for different change types (Loss, Refugia or Gain from climate change), and at the national level and within protected areas, for each of the 19 species of dipterocarps in the Philippines. Current area of suitable habitat would be Loss plus Refugia for any RCP

|  | Future Climate Scenario (RCP 4.5) | | | | | | | | | | | | | | | | | | | | | | |
| --- | --- | --- | --- | --- | --- | --- | --- | --- | --- | --- | --- | --- | --- | --- | --- | --- | --- | --- | --- | --- | --- | --- | --- |
|  | Without Land Cover Correction | | | | | | | | | | |  | With Land Cover Correction | | | | | | | | | | |
|  | National Level | | | | |  | Within Protected Areas | | | | |  | National Level | | | | |  | Within Protected Areas | | | | |
|  | Loss |  | Refugia |  | Gain |  | Loss |  | Refugia |  | Gain |  | Loss |  | Refugia |  | Gain |  | Loss |  | Refugia |  | Gain |
| *D. gracilis* | 52849 |  | 146696 |  | 6986 |  | 4481 |  | 31104 |  | 1831 |  | 10603 |  | 54445 |  | 4193 |  | 1407 |  | 20045 |  | 1237 |
| *D. grandiflorus* | 23088 |  | 192946 |  | 7789 |  | 635 |  | 33719 |  | 2500 |  | 4104 |  | 58748 |  | 5062 |  | 217 |  | 19920 |  | 1840 |
| *D. hasseltii* | 10199 |  | 186600 |  | 11 |  | 843 |  | 31975 |  | 3 |  | 1828 |  | 54260 |  | 8 |  | 466 |  | 19166 |  | 2 |
| *D. kunstleri* | 3284 |  | 28090 |  | 3267 |  | 50 |  | 1687 |  | 599 |  | 810 |  | 7287 |  | 1071 |  | 25 |  | 535 |  | 352 |
| *D. validus* | 1 |  | 54837 |  | 46748 |  | 0 |  | 4702 |  | 4890 |  | 0 |  | 10599 |  | 11670 |  | 0 |  | 2424 |  | 2819 |
| *H. acuminata* | 32235 |  | 28066 |  | 16774 |  | 4779 |  | 7156 |  | 4743 |  | 7906 |  | 14569 |  | 9857 |  | 2862 |  | 5042 |  | 3444 |
| *H. malibato* | 12342 |  | 63831 |  | 21873 |  | 1717 |  | 13757 |  | 4284 |  | 3982 |  | 24199 |  | 9287 |  | 1176 |  | 9284 |  | 2879 |
| *H. plagata* | 4265 |  | 167192 |  | 3260 |  | 465 |  | 33188 |  | 221 |  | 1248 |  | 62480 |  | 669 |  | 249 |  | 20967 |  | 118 |
| *S. almon* | 21603 |  | 172965 |  | 3029 |  | 1336 |  | 26989 |  | 1000 |  | 3961 |  | 54802 |  | 1579 |  | 669 |  | 17078 |  | 785 |
| *S. assamica* | 40712 |  | 50667 |  | 6083 |  | 7690 |  | 11686 |  | 1326 |  | 11544 |  | 21834 |  | 2877 |  | 4867 |  | 7562 |  | 744 |
| *S. astylosa* | 7969 |  | 2195 |  | 1666 |  | 913 |  | 505 |  | 428 |  | 2669 |  | 1252 |  | 1288 |  | 643 |  | 374 |  | 334 |
| *S. contorta* | 15070 |  | 112010 |  | 5995 |  | 1077 |  | 26683 |  | 2058 |  | 2209 |  | 40263 |  | 3924 |  | 418 |  | 17256 |  | 1594 |
| *S. guiso* | 48615 |  | 122616 |  | 23355 |  | 4308 |  | 24627 |  | 6310 |  | 10812 |  | 45705 |  | 8342 |  | 2405 |  | 15725 |  | 3680 |
| *S. hopeifolia* | 21253 |  | 12438 |  | 3774 |  | 1369 |  | 1475 |  | 602 |  | 6088 |  | 4813 |  | 1925 |  | 763 |  | 740 |  | 450 |
| *S. ovata* | 10043 |  | 10250 |  | 6691 |  | 718 |  | 1926 |  | 2338 |  | 3317 |  | 5005 |  | 4218 |  | 386 |  | 1568 |  | 1807 |
| *S. palosapis* | 24080 |  | 17988 |  | 6048 |  | 4659 |  | 5753 |  | 2256 |  | 7437 |  | 12005 |  | 4421 |  | 3103 |  | 4699 |  | 1783 |
| *S. polysperma* | 33346 |  | 78171 |  | 15013 |  | 4286 |  | 19330 |  | 3058 |  | 6688 |  | 37587 |  | 6860 |  | 2590 |  | 13907 |  | 1856 |
| *S. virescens* | 6522 |  | 5263 |  | 4476 |  | 372 |  | 157 |  | 384 |  | 2352 |  | 2834 |  | 2212 |  | 217 |  | 28 |  | 191 |
| *V. pachyphylla* | 12495 |  | 5051 |  | 3940 |  | 796 |  | 690 |  | 1156 |  | 2880 |  | 2783 |  | 2077 |  | 470 |  | 479 |  | 843 |
|  | | | | | | | | | | | | | | | | | | | | | | | |
|  | Future Climate Scenario (RCP 8.5) | | | | | | | | | | | | | | | | | | | | | | |
|  | Without Land Cover Correction | | | | | | | | | | |  | With Land Cover Correction | | | | | | | | | | |
|  | National Level | | | | |  | Within Protected Areas | | | | |  | National Level | | | | |  | Within Protected Areas | | | | |
|  | Loss |  | Refugia |  | Gain |  | Loss |  | Refugia |  | Gain |  | Loss |  | Refugia |  | Gain |  | Loss |  | Refugia |  | Gain |
| *D. gracilis* | 94638 |  | 104908 |  | 10566 |  | 11041 |  | 24544 |  | 2900 |  | 20373 |  | 44675 |  | 6680 |  | 4297 |  | 17155 |  | 2026 |
| *D. grandiflorus* | 28742 |  | 187292 |  | 12116 |  | 924 |  | 33430 |  | 3682 |  | 5323 |  | 57529 |  | 7995 |  | 321 |  | 19816 |  | 2781 |
| *D. hasseltii* | 8346 |  | 188453 |  | 314 |  | 436 |  | 32382 |  | 81 |  | 1025 |  | 55063 |  | 199 |  | 193 |  | 19440 |  | 57 |
| *D. kunstleri* | 5092 |  | 26282 |  | 7421 |  | 95 |  | 1642 |  | 1396 |  | 1069 |  | 7028 |  | 2479 |  | 53 |  | 506 |  | 879 |
| *D. validus* | 3 |  | 54835 |  | 70339 |  | 0 |  | 4702 |  | 8938 |  | 0 |  | 10599 |  | 20555 |  | 0 |  | 2424 |  | 5149 |
| *H. acuminata* | 43944 |  | 16357 |  | 23050 |  | 7614 |  | 4321 |  | 6758 |  | 12588 |  | 9886 |  | 14090 |  | 4583 |  | 3321 |  | 5066 |
| *H. malibato* | 17522 |  | 58650 |  | 26709 |  | 1950 |  | 13524 |  | 6404 |  | 5411 |  | 22771 |  | 13376 |  | 1224 |  | 9235 |  | 4510 |
| *H. plagata* | 9563 |  | 161895 |  | 3045 |  | 1093 |  | 32560 |  | 278 |  | 1936 |  | 61793 |  | 957 |  | 440 |  | 20776 |  | 177 |
| *S. almon* | 22965 |  | 171603 |  | 5668 |  | 1129 |  | 27196 |  | 2086 |  | 4656 |  | 54106 |  | 3034 |  | 406 |  | 17340 |  | 1530 |
| *S. assamica* | 56636 |  | 34743 |  | 6359 |  | 10557 |  | 8819 |  | 1474 |  | 16760 |  | 16618 |  | 3385 |  | 6755 |  | 5673 |  | 900 |
| *S. astylosa* | 9385 |  | 780 |  | 1653 |  | 1211 |  | 207 |  | 561 |  | 3432 |  | 489 |  | 1394 |  | 878 |  | 139 |  | 474 |
| *S. contorta* | 21385 |  | 105694 |  | 9661 |  | 1994 |  | 25766 |  | 3059 |  | 3545 |  | 38927 |  | 6173 |  | 931 |  | 16743 |  | 2289 |
| *S. guiso* | 49611 |  | 121619 |  | 18252 |  | 6460 |  | 22474 |  | 6107 |  | 14090 |  | 42426 |  | 9865 |  | 3599 |  | 14531 |  | 4260 |
| *S. hopeifolia* | 26210 |  | 7481 |  | 3941 |  | 1789 |  | 1054 |  | 734 |  | 7830 |  | 3071 |  | 2095 |  | 1042 |  | 460 |  | 463 |
| *S. ovata* | 13629 |  | 6663 |  | 6595 |  | 1166 |  | 1478 |  | 2237 |  | 4738 |  | 3584 |  | 4407 |  | 730 |  | 1224 |  | 1768 |
| *S. palosapis* | 31515 |  | 10554 |  | 9325 |  | 6725 |  | 3687 |  | 3710 |  | 11094 |  | 8347 |  | 7298 |  | 4613 |  | 3189 |  | 3059 |
| *S. polysperma* | 51778 |  | 59739 |  | 17197 |  | 7397 |  | 16219 |  | 3919 |  | 11967 |  | 32308 |  | 9230 |  | 4547 |  | 11950 |  | 2511 |
| *S. virescens* | 8730 |  | 3055 |  | 6670 |  | 392 |  | 138 |  | 605 |  | 3622 |  | 1563 |  | 3339 |  | 211 |  | 34 |  | 315 |
| *V. pachyphylla* | 14147 |  | 3399 |  | 2689 |  | 1004 |  | 482 |  | 1061 |  | 3577 |  | 2086 |  | 1741 |  | 584 |  | 364 |  | 845 |

**Table S7.** The MESS analysis and result for the current and two future climate scenarios (RCP 4.5 and RCP 8.5) for 2070, as a percentage for Total (pixels with MESS scores >0) and High (pixels with MESS scores >10)

|  | Total | | | | |  | High | | | | |
| --- | --- | --- | --- | --- | --- | --- | --- | --- | --- | --- | --- |
|  | Current |  | RCP 4.5 |  | RCP 8.5 |  | Current |  | RCP 4.5 |  | RCP 8.5 |
| *D. gracilis* | 0.1% |  | 9.4% |  | 9.4% |  | 0.0% |  | 0.0% |  | 0.0% |
| *D. grandiflorus* | 0.4% |  | 13.2% |  | 13.2% |  | 0.0% |  | 0.2% |  | 0.2% |
| *D. hasseltii* | 0.1% |  | 9.4% |  | 9.4% |  | 0.0% |  | 0.0% |  | 0.0% |
| *D. kunstleri* | 0.2% |  | 14.1% |  | 14.1% |  | 0.0% |  | 0.0% |  | 0.0% |
| *D. validus* | 0.3% |  | 17.7% |  | 17.7% |  | 0.0% |  | 0.2% |  | 0.2% |
| *H. acuminata* | 0.0% |  | 15.2% |  | 15.2% |  | 0.0% |  | 0.0% |  | 0.0% |
| *H. malibato* | 0.1% |  | 14.2% |  | 14.2% |  | 0.0% |  | 0.0% |  | 0.0% |
| *H. plagata* | 0.1% |  | 20.1% |  | 20.1% |  | 0.0% |  | 0.0% |  | 0.0% |
| *S. almon* | 0.0% |  | 19.9% |  | 19.9% |  | 0.0% |  | 0.0% |  | 0.0% |
| *S. assamica* | 1.2% |  | 15.2% |  | 15.2% |  | 0.0% |  | 0.0% |  | 0.0% |
| *S. astylosa* | 0.1% |  | 27.6% |  | 27.6% |  | 0.0% |  | 0.0% |  | 0.0% |
| *S. contorta* | 0.1% |  | 17.6% |  | 17.6% |  | 0.0% |  | 0.1% |  | 0.1% |
| *S. guiso* | 0.1% |  | 9.9% |  | 9.9% |  | 0.0% |  | 0.1% |  | 0.1% |
| *S. hopeifolia* | 0.2% |  | 20.0% |  | 20.0% |  | 0.0% |  | 0.1% |  | 0.1% |
| *S. ovata* | 0.0% |  | 10.1% |  | 10.1% |  | 0.0% |  | 0.0% |  | 0.0% |
| *S. palosapis* | 0.1% |  | 18.9% |  | 18.9% |  | 0.0% |  | 0.1% |  | 0.1% |
| *S. polysperma* | 0.3% |  | 18.8% |  | 18.8% |  | 0.0% |  | 0.0% |  | 0.0% |
| *S. virescens* | 0.1% |  | 18.7% |  | 18.7% |  | 0.0% |  | 0.1% |  | 0.1% |
| *V. pachyphylla* | 15.6% |  | 33.3% |  | 33.3% |  | 2.0% |  | 3.3% |  | 3.3% |


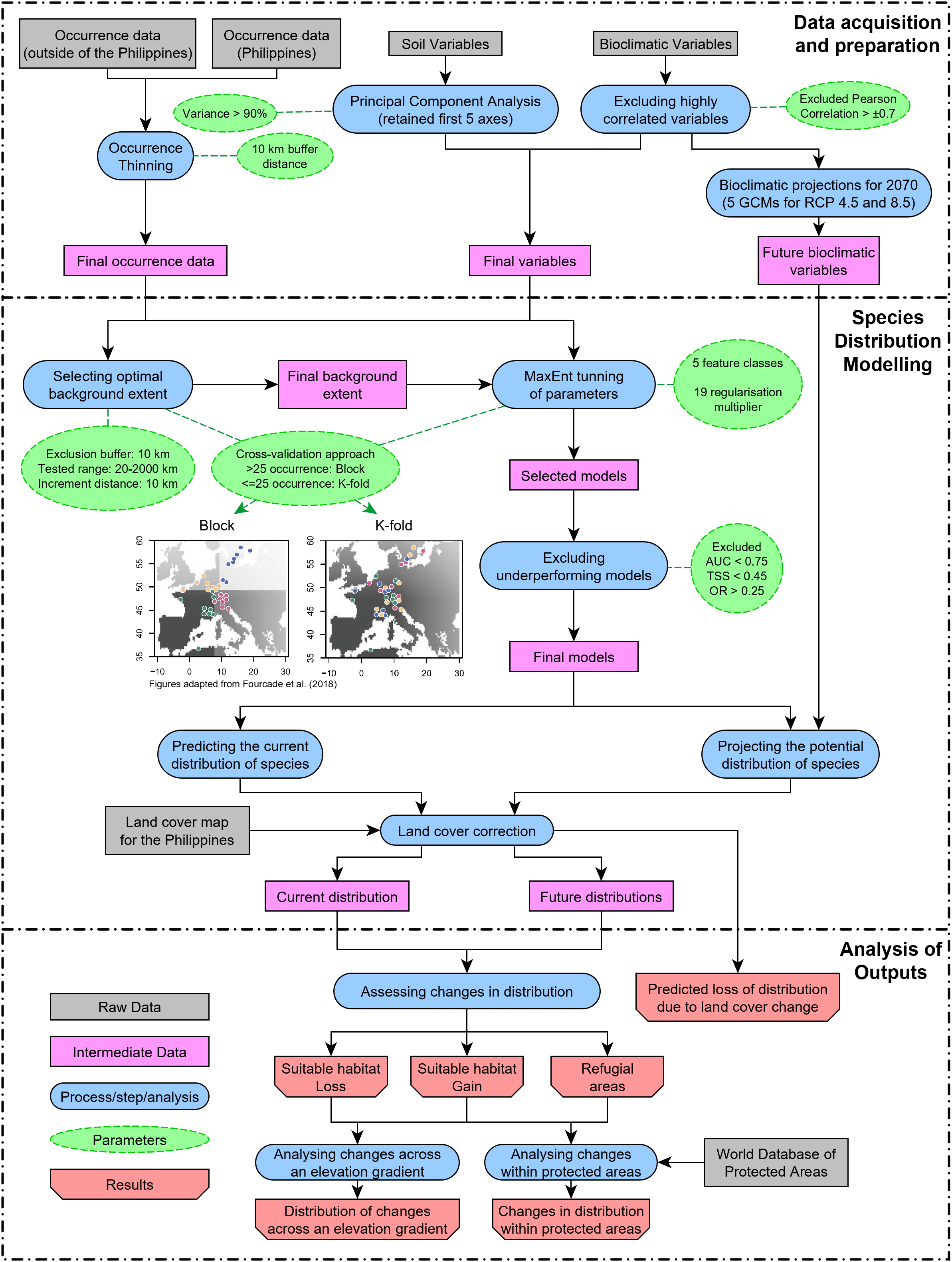


**Figure S1**. The entire workflow for the study broken into three main sections: 1) data acquisition and preparation; 2) species distribution modelling; and 3) analysis of outputs. Flowchart was created by Sean E. H. Pang using yEd (https://www.yworks.com/products/yed)


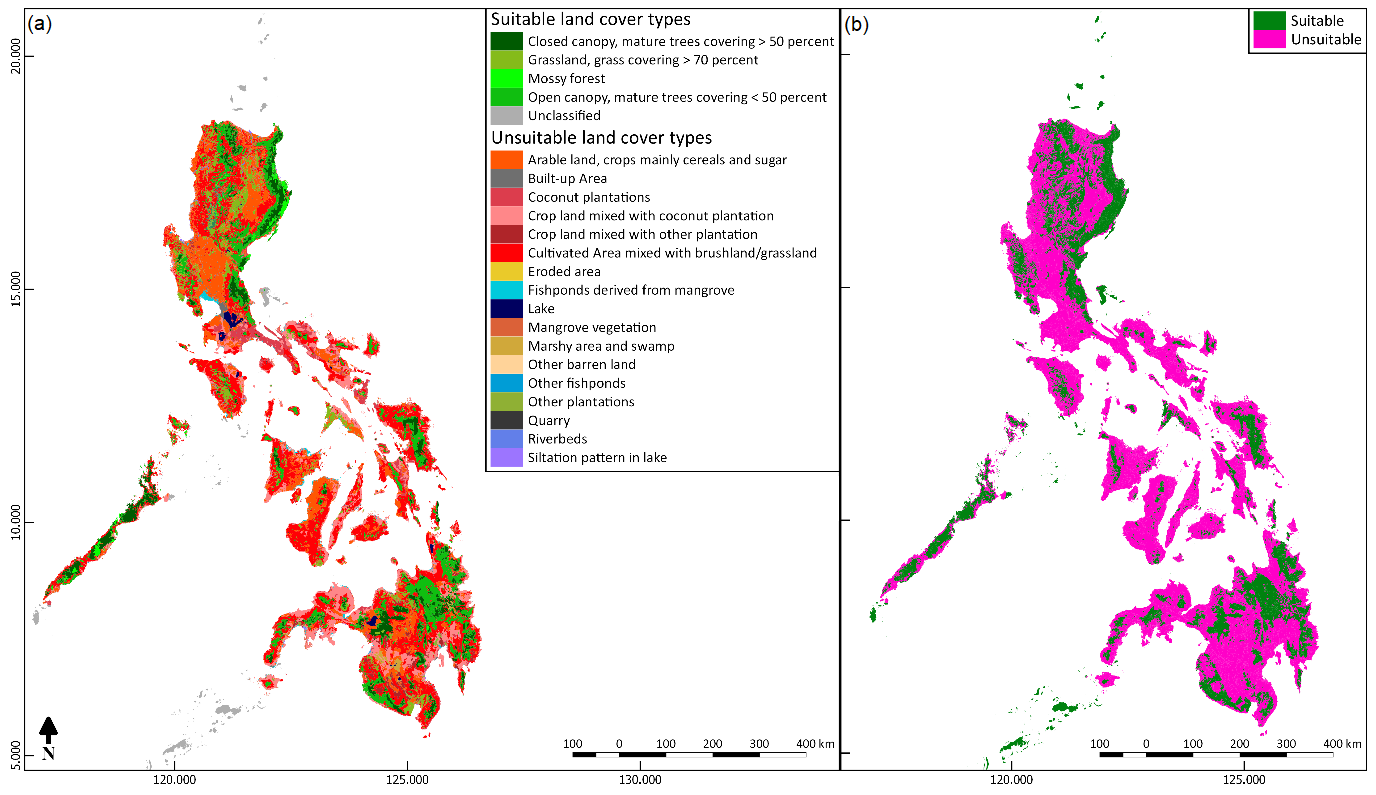
**Figure S2.** Map of the Philippines with the different land cover types and their designated suitability. (a) Land cover types of the Philippines (www.philgis.org). (b) Land cover types reclassified as suitable or unsuitable (see Table S4). Maps were created using QGIS^[1]^.

**Figure S3.** Species-specific scaled elevation distribution—with LCC—of dipterocarps in the Philippines for the current, and two future climate scenarios (RCP 4.5 and RCP 8.5). The figure was created in R using the ggplot2 package^[2,3]^.


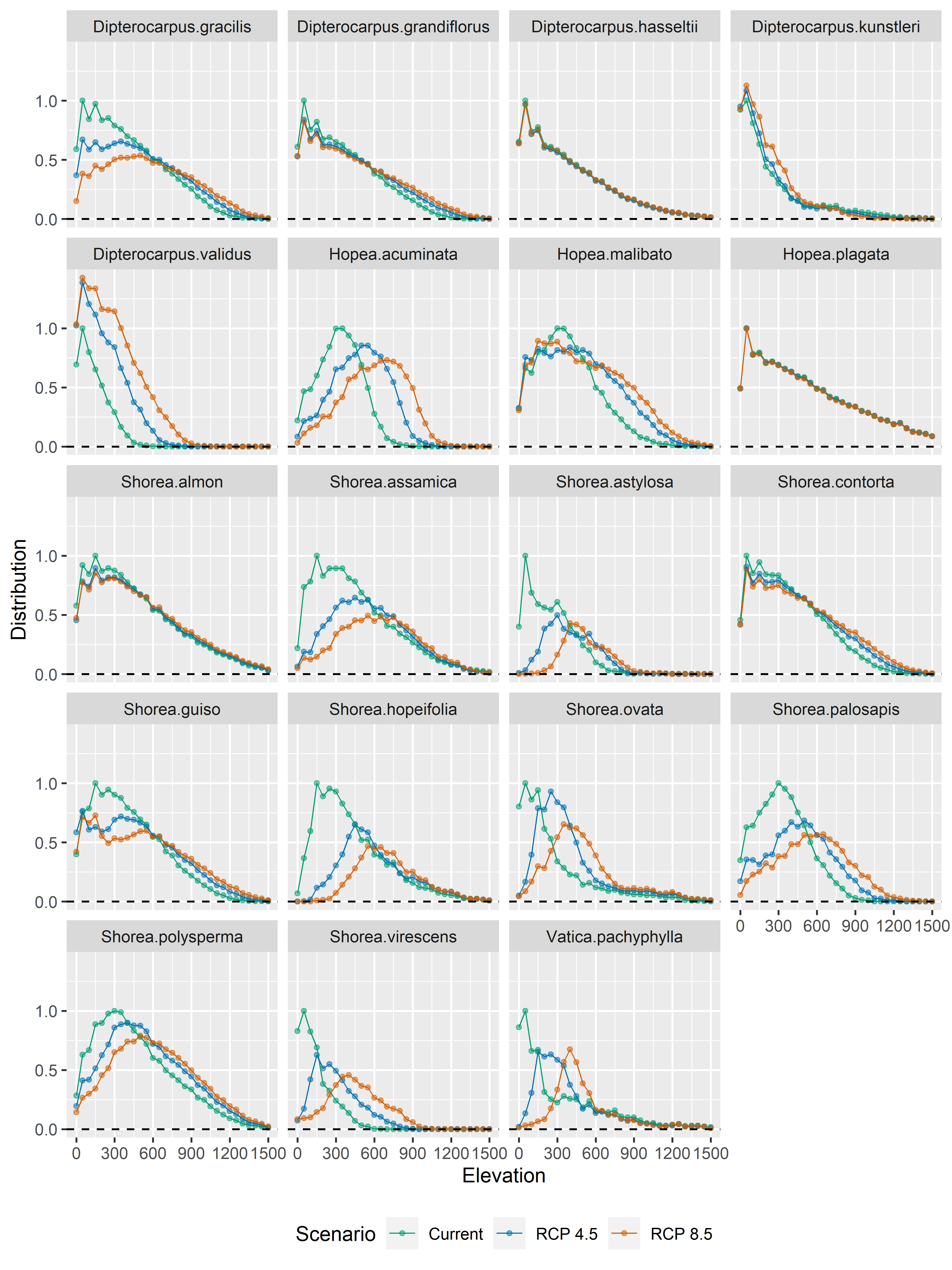

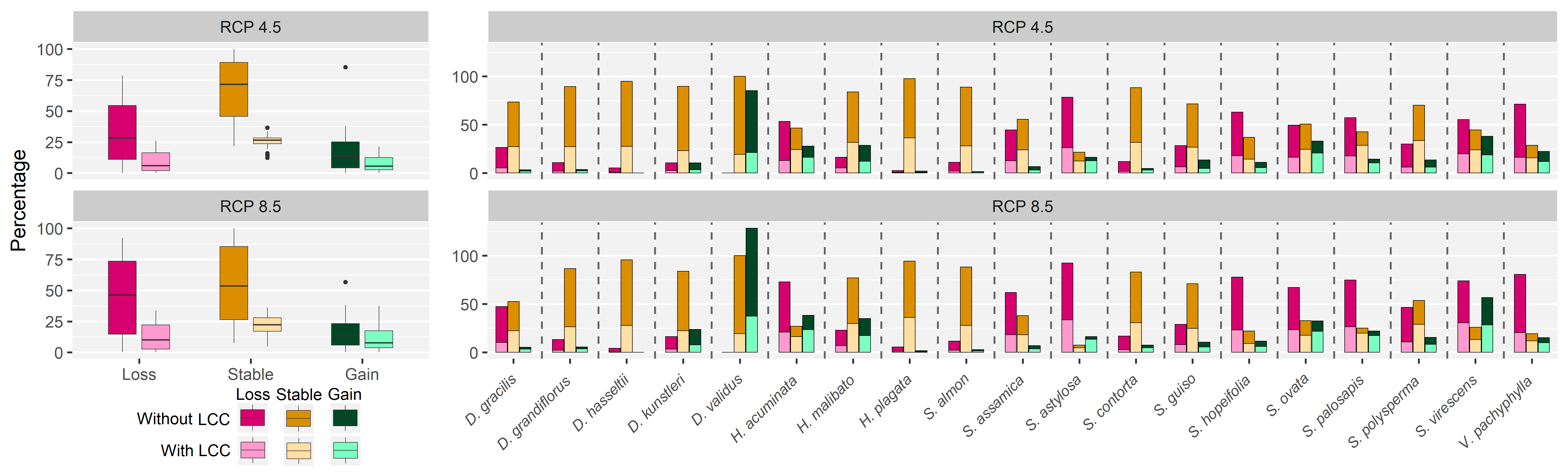


**Figure S4.** The percentage change of suitable habitat for loss, stable and gain from LCC and climate change for Philippine dipterocarps across the Philippines, under two future climate scenarios (RCP 4.5 and RCP 8.5 for 2070), (a) all species combined, and (b) for individual species. Changes to suitable habitat were either loss, stable or gain, indicated by Magenta, Orange or Green hues, respectively. Darker hues indicated climate-induced changes in suitable habitat without LCC, which included areas considered unsuitable due to anthropogenic land use; while the lighter hues indicated climate-induced changes with LCC, which excluded areas considered unsuitable due to anthropogenic land use. The figure was created in R using the ggplot2 package^[2,3]^.


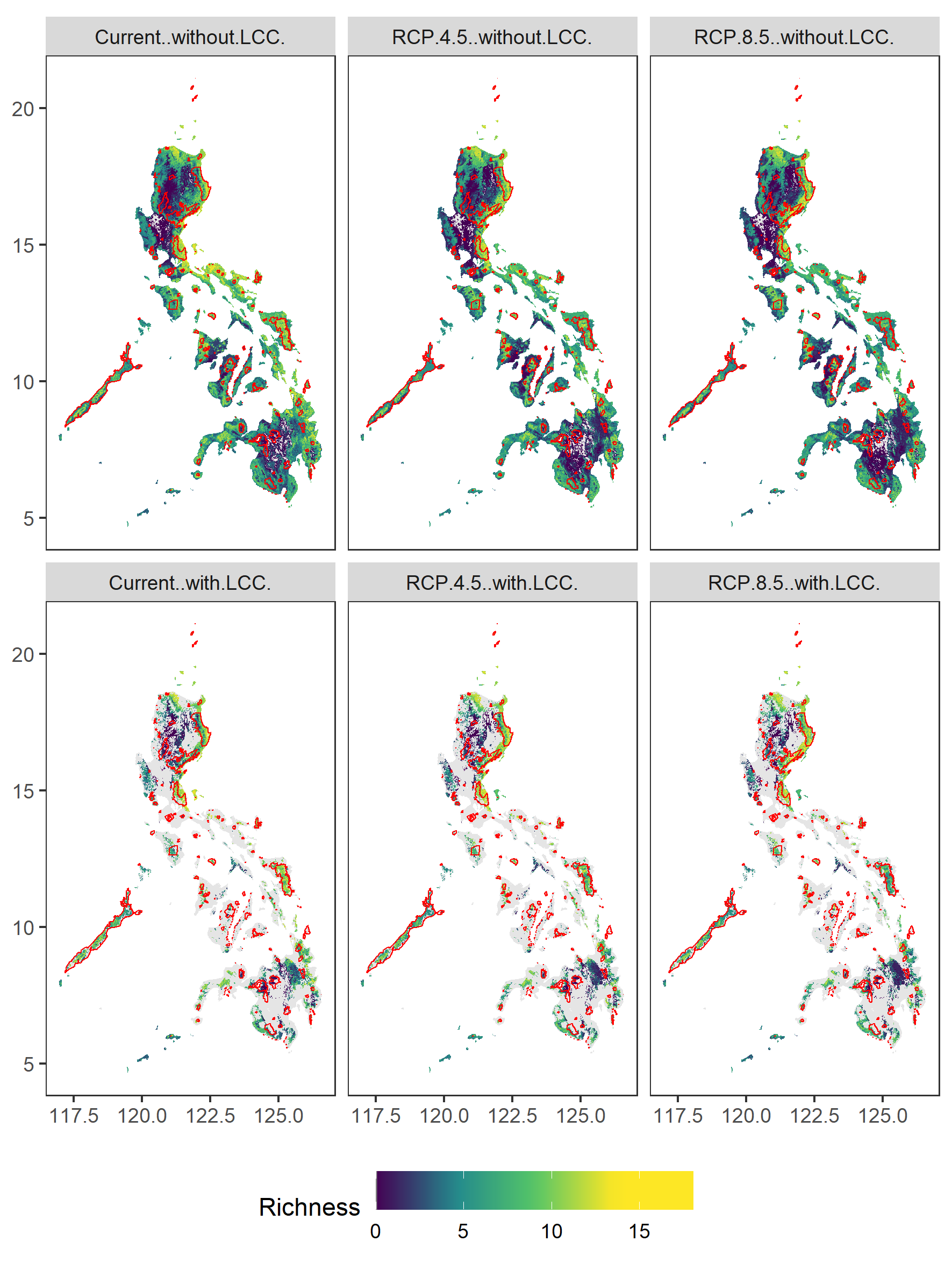


**Figure S5.** Stack distribution of suitable habitat for the 19 species of dipterocarps in the Philippines under the current and two future (RCP 4.5 and RCP 8.5) climate scenarios (i.e., distribution of stable with gain for future scenarios), without and with LCC applied. Protected areas are demarcated in red. The maps were created in R using the raster and ggplot2 package^[2–4]^.


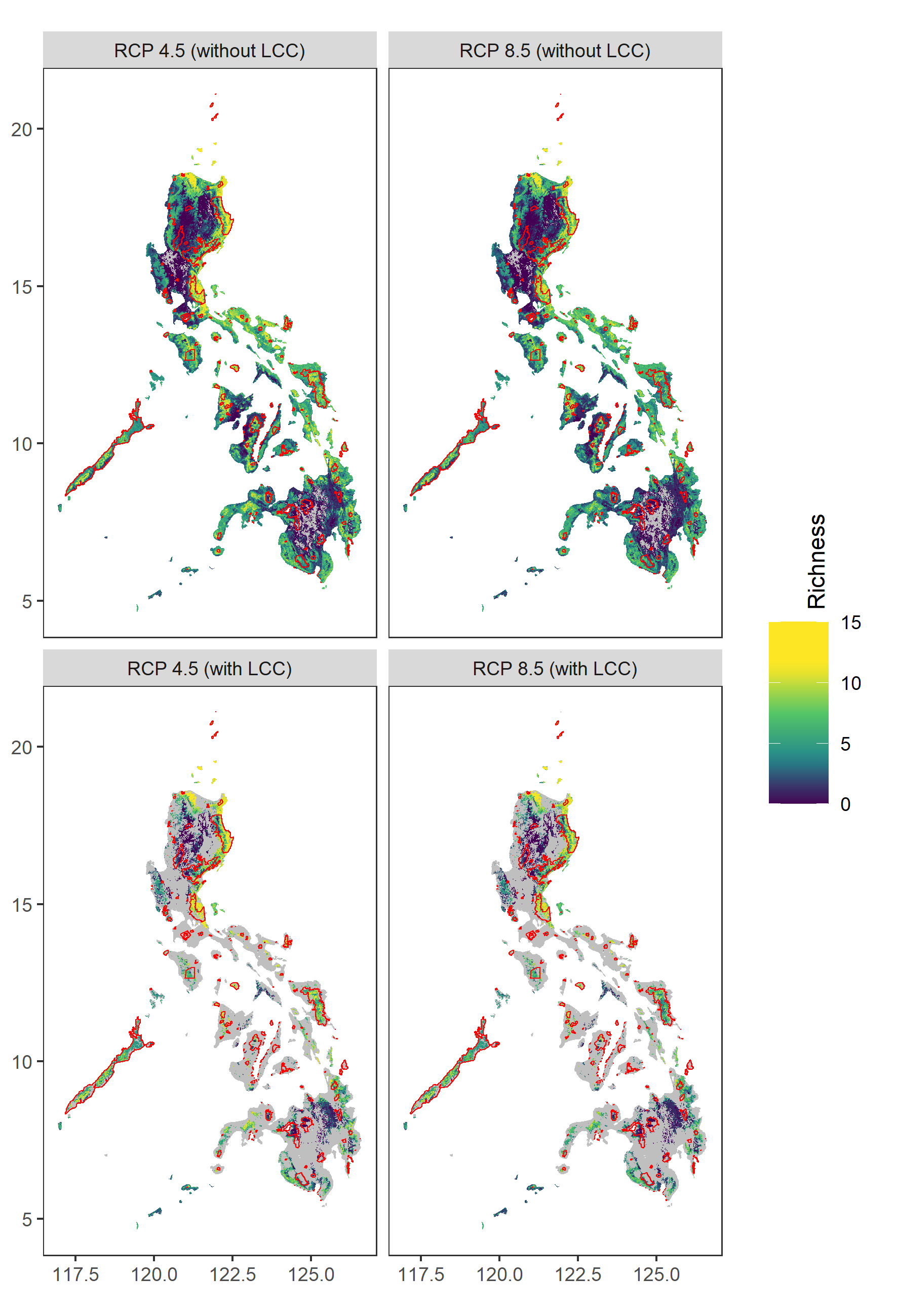


**Figure S6.** Stack suitable habitat distribution of stable (i.e., climatic macrorefugia) for the 19 species of dipterocarps in the Philippines under two future (RCP 4.5 and RCP 8.5) climate scenarios, without and with LCC applied. Protected areas are demarcated in red. The maps were created in R using the raster and ggplot2 package^[2–4]^.


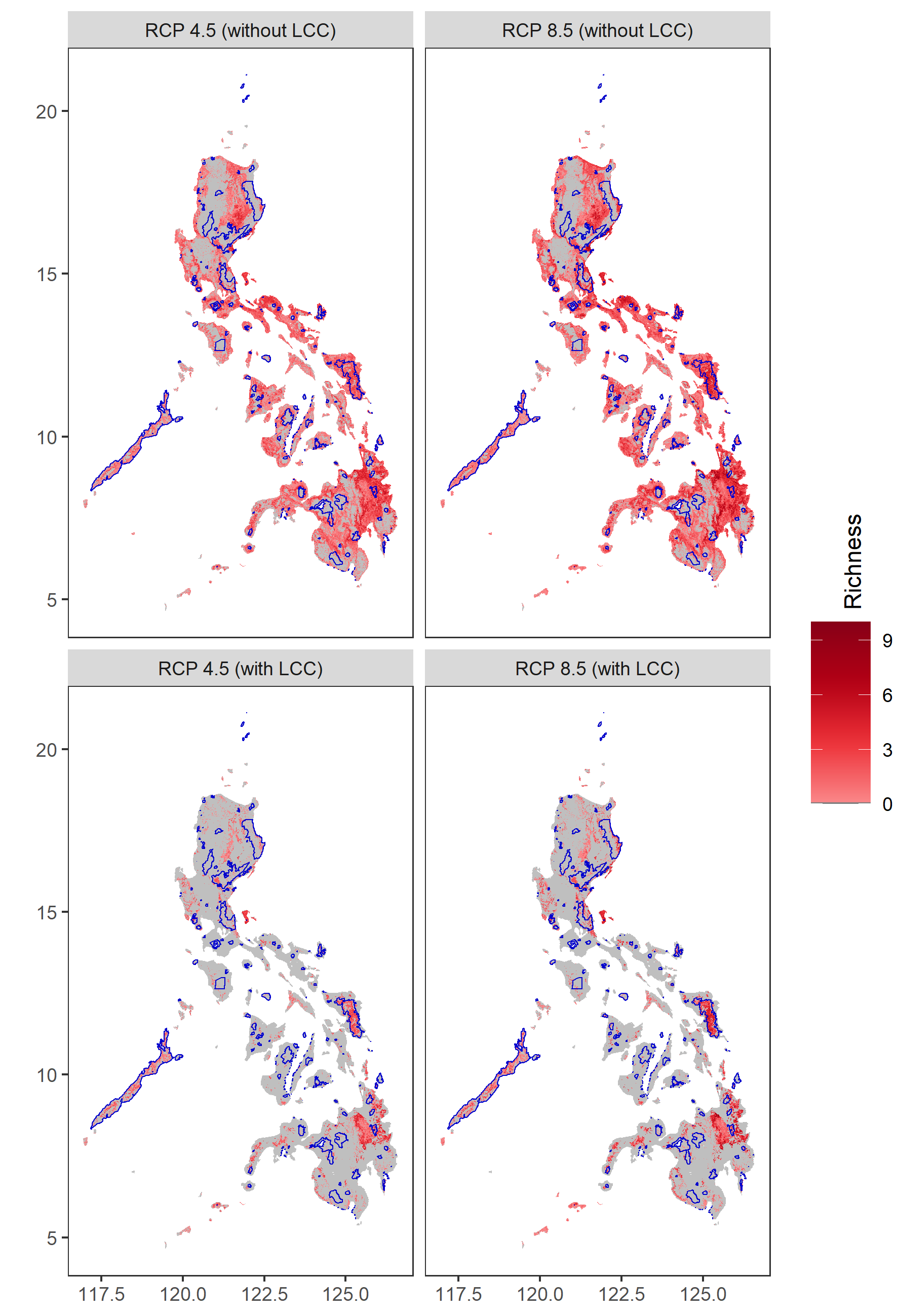


**Figure S7.** Stack suitable habitat distribution of loss for the 19 species of dipterocarps in the Philippines under two future (RCP 4.5 and RCP 8.5) climate scenarios, without and with LCC applied. Protected areas are demarcated in blue. The maps were created in R using the raster and ggplot2 package^[2–4]^.


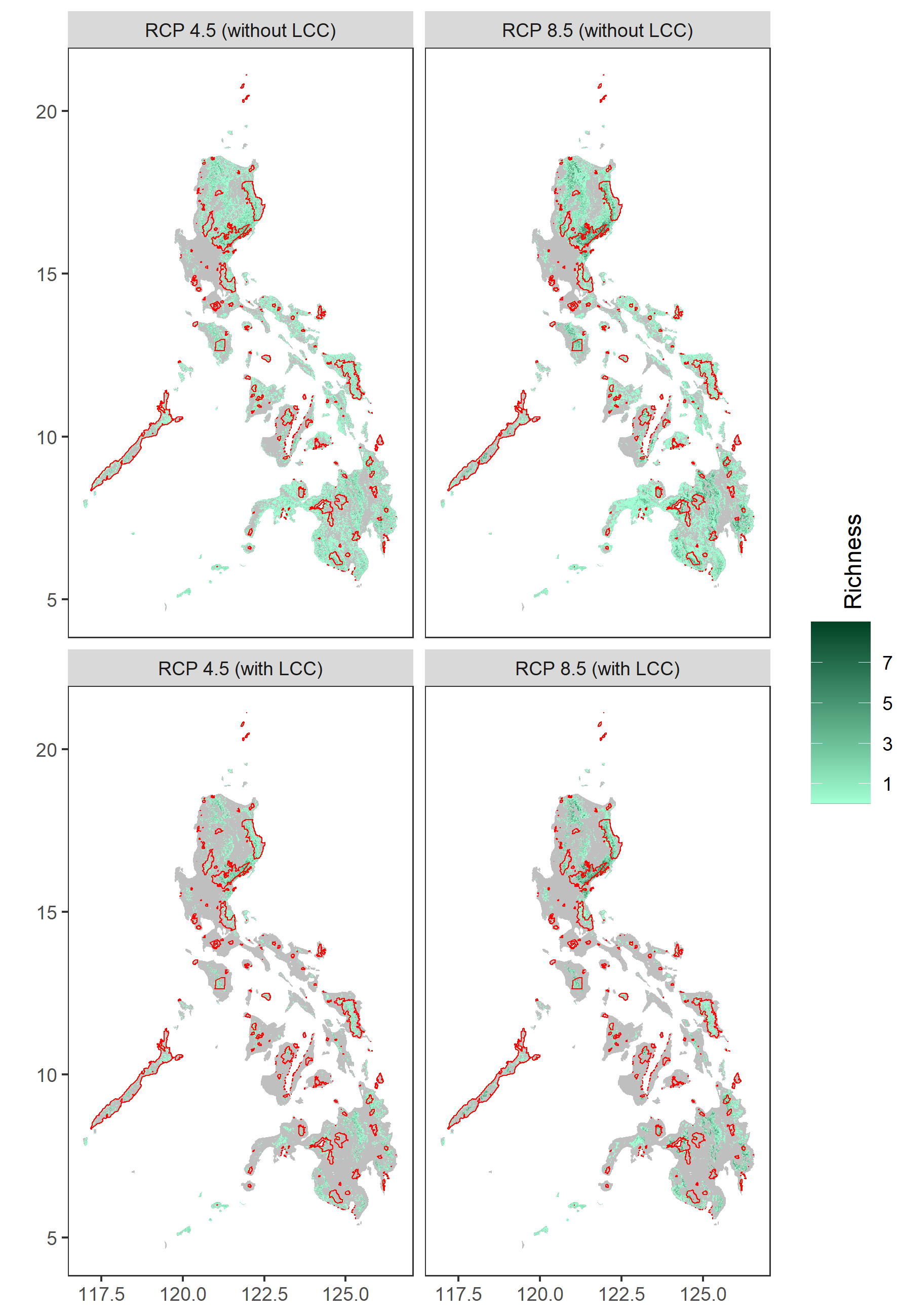


**Figure S8.** Stack suitable habitat distribution of gain for the 19 species of dipterocarps in the Philippines under two future (RCP 4.5 and RCP 8.5) climate scenarios, without and with LCC applied. Protected areas are demarcated in red. The maps were created in R using the raster and ggplot2 package^[2–4]^.

**References**

1. Quantum, G.I.S. Development Team, 2017 Quantum GIS Geographic Information System. Open Source Geospatial Foundation Project. [Accessed 2017 Sep 16].

2. R Core Team. R: A language and environment for statistical computing. R Found. Stat. Comput. Vienna Austria **55**, 275–286 (2013).

3. Wickham, H. ggplot2: elegant graphics for data analysis. (springer, 2016).

4. Hijmans, R.J. & Etten, J.V. Geographic analysis and modeling with raster data. R Package Version **2**, 1–25 (2012).
